# IL-1β-activated PI3K/AKT and MEK/ERK pathways coordinately promote induction of partial epithelial–mesenchymal transition

**DOI:** 10.1186/s12964-024-01775-8

**Published:** 2024-08-08

**Authors:** Yosuke Tabei, Yoshihiro Nakajima

**Affiliations:** Health and Medical Research Institute, National Institute of Advanced Industrial Science and Technology (AIST), 2217-14 Hayashi-Cho, Takamatsu, Kagawa 761-0395 Japan

**Keywords:** Interleukin-1β, Epithelial–mesenchymal transition, PI3K/AKT pathway, MEK/ERK pathway

## Abstract

Epithelial–mesenchymal transition (EMT) is a cellular process in embryonic development, wound healing, organ fibrosis, and cancer metastasis. Previously, we and others have reported that proinflammatory cytokine interleukin-1β (IL-1β) induces EMT. However, the exact mechanisms, especially the signal transduction pathways, underlying IL-1β-mediated EMT are not yet completely understood. Here, we found that IL-1β stimulation leads to the partial EMT-like phenotype in human lung epithelial A549 cells, including the gain of mesenchymal marker (vimentin) and high migratory potential, without the complete loss of epithelial marker (E-cadherin). IL-1β-mediated partial EMT induction was repressed by PI3K inhibitor LY294002, indicating that the PI3K/AKT pathway plays a significant role in the induction. In addition, ERK1/2 inhibitor FR180204 markedly inhibited the IL-1β-mediated partial EMT induction, demonstrating that the MEK/ERK pathway was also involved in the induction. Furthermore, we found that the activation of the PI3K/AKT and MEK/ERK pathways occurred downstream of the epidermal growth factor receptor (EGFR) pathway and the IL-1 receptor (IL-1R) pathway, respectively. Our findings suggest that the PI3K/AKT and MEK/ERK pathways coordinately promote the IL-1β-mediated partial EMT induction. The inhibition of not one but both pathways is expected yield clinical benefits by preventing partial EMT-related disorders such as organ fibrosis and cancer metastasis.

**Supplementary Information:**

The online version contains supplementary material available at 10.1186/s12964-024-01775-8.

## Background

Epithelial–mesenchymal transition (EMT) is a cellular process in which fully differentiated epithelial cells lose such characteristics as cell–cell adhesion and apical–basal polarity and acquire mesenchymal properties including motility and invasiveness [1]. Whereas EMT is a well-regulated, normal cellular process in embryonic development and wound healing, promoting tissue and organ formation, its dysregulation leads to pathological conditions, such as tissue fibrosis and cancer progression and metastasis [2]. Indeed, several studies on human specimens have disclosed that the EMT process is involved in fibrotic diseases of multiple organs including lung, kidney, and liver [3]. In addition, studies on tumor samples have indicated that EMT is activated in primary epithelial tumors [4].

The hallmarks of EMT are the downregulation of epithelial markers such as E-cadherin and occludin and the upregulation of mesenchymal markers such as vimentin and fibronectin [5]. EMT is activated by various environmental stimuli including growth factors, such as transforming growth factor-β (TGF-β) and epidermal growth factor (EGF), hypoxia, and extracellular matrix components, that act through EMT-related transcriptional factors to repress epithelial gene expression and induce mesenchymal gene expression [6, 7]. EMT is generally considered a binary process involving a complete conversion from the epithelial state into the mesenchymal state. However, it become apparent that EMT also encompasses a range of hybrid states, a phenotype referred to as partial EMT (also known as hybrid E/M or intermediate EMT) [8, 9]. Partial EMT is defined by an incomplete loss of epithelial markers and an incomplete gain of mesenchymal markers [10]. A partial EMT state has been noted in association with many developmental processes, wound healing, fibrosis, and cancer progression [11]. For example, tubular epithelial cells undergo partial EMT by expressing both epithelial and mesenchymal markers, thereby contributing to the development and progression of renal fibrosis [12]. In addition, lung epithelial cells from patients presenting with idiopathic pulmonary fibrosis co-express epithelial and mesenchymal markers [13]. Notably, cancer cells in the partial EMT state show greater tumor-initiating potential, therapeutic resistance, and apoptosis resistance than complete epithelial or mesenchymal cells [14]. In addition, cancer cells in the partial EMT state tend to adapt to the metastatic microenvironment, resulting in an increased risk of metastasis [15]. The occurrence of chemoresistant cancer cells in the partial EMT state was observed in metastatic breast cancer patients receiving chemotherapy, contributing to poor clinical outcomes [16]. Although many partial EMT states have been found in clinical samples, their physiological functions are still not fully understood. Therefore, elucidating the mechanism underlying EMT will usher in new therapies to improve overall survival in patients diagnosed with fibrosis, cancer, and associated diseases.

Inflammation is a protective response of the host to infection and tissue damage, which can prevent the spread of pathogens or promote tissue repair [17]. Inflammation is usually resolved in a time-dependent manner and therefore beneficial to the host. On the other hand, dysregulated inflammation often gives rise to such pathological conditions as fibrosis and cancer [18]. In addition, increasing evidence has emphasized a link between inflammation and EMT, which is correlated with tumor aggressiveness and poor outcomes [19]. For example, tumor necrosis factor-α (TNF-α), a proinflammatory cytokine predominantly produced by macrophages, contributes to EMT induction in tumor cells and invasion through the activation of the nuclear factor kappa-B (NF-κB) and protein kinase B (AKT) signaling pathways [20]. Interleukin (IL)-6, a key inflammatory cytokine, is also able to induce EMT through the activation of the janus kinase (JAK)/signal transducer and activator of transcription 3 (STAT3) signaling pathway [21]. IL-8, a chemotactic factor that attracts neutrophils, basophils, and T cells during inflammation, is involved in the EMT process via the activation of signaling cascades including extracellular signal-regulated protein kinase (ERK), JAK2/STAT3, and phosphoinositide 3-kinase (PI3K)/AKT signaling pathways [22–24].

Recently, we reported that IL-1β, which is released from macrophages stimulated with nanoparticles that cause pulmonary fibrosis, can induce EMT in alveolar epithelial cells [25]. Although one of the most well-studied proinflammatory cytokines and not generally expressed in healthy cells, IL-1β is rapidly released to orchestrate early response of tissue to damage in the lung and other organs. Although knowledge of IL-1β-mediated EMT is limited, some reports have suggested a link between IL-1β stimulation and EMT induction. Li et al. showed that IL-1β treatment promotes EMT in colon cancer cells [26]. In addition, oral squamous cell carcinoma treated with IL-1β led to decreased E-cadherin levels and increased vimentin levels, which are characteristic of EMT [27]. Li et al. recently disclosed that chronic exposure of non-small cell lung cancer (NSCLC) to IL-1β-induced EMT via epigenetic modifications [28]. However, the exact mechanisms, especially the signal transduction pathways, underlying IL-1β-mediated EMT are not yet completely understood.

The purpose of this study was to understand the inductive mechanism of IL-1β-mediated EMT, focusing on intracellular signal transduction pathways. We showed that the coordinated activation of PI3K/AKT and mitogen-activated and extracellular signal-regulated kinase kinase (MEK)/ERK signaling pathways is required for the induction of IL-1β-mediated EMT. The former is activated downstream of the epidermal growth factor receptor (EGFR) signaling cascade, whereas the latter is activated downstream of the IL-1 receptor (IL-1R) signaling cascade.

## Methods

### Reagents

Recombinant human IL-1β, TGF-β1, EGF, and AREG were purchased from R&D Systems (Minneapolis, MN). Primary antibodies and chemical inhibitors used in this study are summarized in Tables S1 and S2, respectively. To investigate the effects of inhibitors on the induction of EMT, the cells were pretreated with inhibitors for 4 h and then stimulated with 100 pg/mL IL-1β for an additional 48 h. Selective chemical inhibitors are particularly useful for understanding the function of a single kinase in complex cellular signaling [29]. All chemical inhibitors were dissolved in dimethyl sulfoxide (DMSO; Sigma-Aldrich, Saint Louis, MO), and the final concentration of DMSO was lower than 0.1%.

### Cell culture

In the present study, we used human lung epithelial A549 cells as an in vitro model to explore the signaling pathway triggered by IL-1β, because the cells have been widely used for studying toxicology, pharmacology, and pathology of lung tissue. The A549 cells were purchased from the RIKEN BioResource Research Center (Ibaraki, Japan) and maintained in Dulbecco’s modified Eagle medium (DMEM; Thermo Fisher Scientific, Inc., Waltham, MA) supplemented with 10% heat-inactivated fetal bovine serum (FBS; Sigma-Aldrich), 100 U/mL penicillin, 100 μg/mL streptomycin, and 250 ng/mL amphotericin B (Nacalai Tesque Inc., Kyoto, Japan) at 37 °C in a humidified atmosphere with 5% CO_2_. For the experiments, EMT was induced as described previously [25].

### Cell viability assay

The cells were incubated with various concentrations of chemical inhibitors for 48 h. To determine cell viability, the cells were incubated with WST-1 solution (Takara Bio, Shiga, Japan) at 37 °C for 30 min. The optical density of formazan was measured at 450 nm using a Tecan Infinite M200 (Tecan Group Ltd., Männedorf, Switzerland) and summarized in Fig. S1. Working concentrations of the chemical inhibitors were selected on the basis of the following principals: the selected concentration does not cause > 50% cell death and makes it possible to study the EMT phenotypes after a 48-h treatment.

### Cell morphology determination

Cell morphology was determined as described previously [25]. In brief, the cells were fixed with 4% paraformaldehyde for 15 min, washed with PBS three times, and then stained with 5 μg/mL Hoechst 33342 (Thermo Fisher Scientific, Inc.) and 2 μM CellTracker Red CMTPX Dye (Thermo Fisher Scientific, Inc.) for 30 min. Fluorescence images were captured using a CQ1 Confocal Quantitative Image Cytometer (Yokogawa Electric Corp., Tokyo, Japan). CellPathfinder software (Yokogawa Electric Corp.) was used to measure the aspect ratio.

### Total RNA isolation and quantitative real-time PCR

Total RNA was extracted using an RNeasy Mini Kit (Qiagen, Valencia, CA) following the manufacturer’s instructions. First-strand cDNA was synthesized using a PrimeScript RT Master Mix (Takara Bio). *CDH1*, *VIM*, and *ACTB* mRNA levels were analyzed using the TaqMan Gene Expression Assay (IDs: CDH1, Hs01023895_m1; VIM, Hs00958111_m1; ACTB, Hs99999903_m1). *ACTB* was used as internal control. Target mRNA levels were measured using a Thermal Cycler Dice Real Time System III (Takara Bio). The mRNA expression levels in each sample were normalized to *ACTB* value and then compared with untreated controls.

### Western blot analysis

For western blot analysis, the cells were lysed with RIPA buffer (Thermo Fisher Scientific, Inc.) supplemented with Halt Protease and Phosphatase Inhibitor Cocktail (Thermo Fisher Scientific, Inc.). Protein concentrations were measured using a Pierce BCA Protein Assay Kit (Thermo Fisher Scientific. Inc.). Protein levels were analyzed using the capillary electrophoresis western system Wes (ProteinSimple Co., San Jose, CA) following the manufacturer’s instructions [30, 31]. In brief, the protein samples were mixed with 5 × Fluorescent Master Mix (ProteinSimple Co.) and then heated at 95 °C for 5 min. Next, the heated samples, primary antibodies (Table S1), biotinylated protein ladder (ProteinSimple Co.), horseradish peroxide-conjugated anti-rabbit secondary antibody (ProteinSimple Co.), and luminol-peroxide (ProteinSimple Co.) were loaded into the 12–230 kDa Wes Separation Module (ProteinSimple Co.). Protein levels were analyzed using Compass software for Simple Western (ProteinSimple Co.). Whole images of western blotting are shown in Fig. S2.

### Immunofluorescence analysis

Cells were washed with PBS and fixed with 4% paraformaldehyde (Fujifilm Wako Pure Chemical Co., Osaka, Japan) for 15 min. Then, the cells were washed three times and permeabilized with 0.1% Triton X-100 (Sigma-Aldrich) for 10 min. After permeabilization, the cells were washed three times and blocked with 1% BSA for 1 h. After that, the cells were incubated with primary antibody (Table S1) overnight at 4 °C. The cells were washed three times and incubated for 1 h with goat anti-rabbit IgG Alexa 594 (1:1000 dilution; Thermo Fisher Scientific. Inc.). After washing with PBS, the cells were mounted with ProLong Glass Antifade Mountant with NucBlue Stain (Thermo Fisher Scientific. Inc.). Fluorescence images were obtained on a BZ-X710 fluorescence microscope (Keyence, Osaka, Japan).

### Wound healing assay

For the wound healing assay, double-well culture inserts (ibidi GmbH, Martinsried, Germany) were placed at the bottom of a 24-well plate. Cells were seeded into each chamber of the inserts. After the cells were incubated for 24 h, the inserts were removed to create wounds. Imaging of the wound was performed every hour for 48 h using a Lionheart FX Automated Live Cell Imager (BioTek Instruments, Winooski, VT). Quantification of wound confluence was performed using Gen5 software (BioTek Instruments).

### Phospho-kinase assay

Kinase phosphorylation in A549 cells treated with IL-1β was determined using a Proteome Profiler Human Phospho-Kinase Array Kit (R&D Systems) following the manufacturer’s instructions. Spots were imaged using an ImageQuant LAS 4000 system. The mean density of each spot was measured and quantified using ImageQuant TL software following a previous report [25]. In brief, the reciprocal was calculated and then corrected for the value of the background density. Then, the integrated density of each pair of phospho-kinases was expressed as a proportion of the integrated density of the reference spots. Values for each row were normalized to have the highest phosphor-kinase response as 1.

### Statistical analysis

All assays were conducted in triplicate at least. Data are expressed as means with standard deviations (SDs). Comparisons between two groups were performed with the Student’s *t*-test and those between more than two groups, with one-way analysis of variance (ANOVA) or two-way ANOVA followed by Tukey’s multiple comparison test. GraphPad Prism 8.4.3 was used for statistical analysis, and a *P* value less than 0.05 was considered statistically significant.

## Results

### IL-1β induces partial EMT-like phenotype in A549 cells

**Fig. 1:**
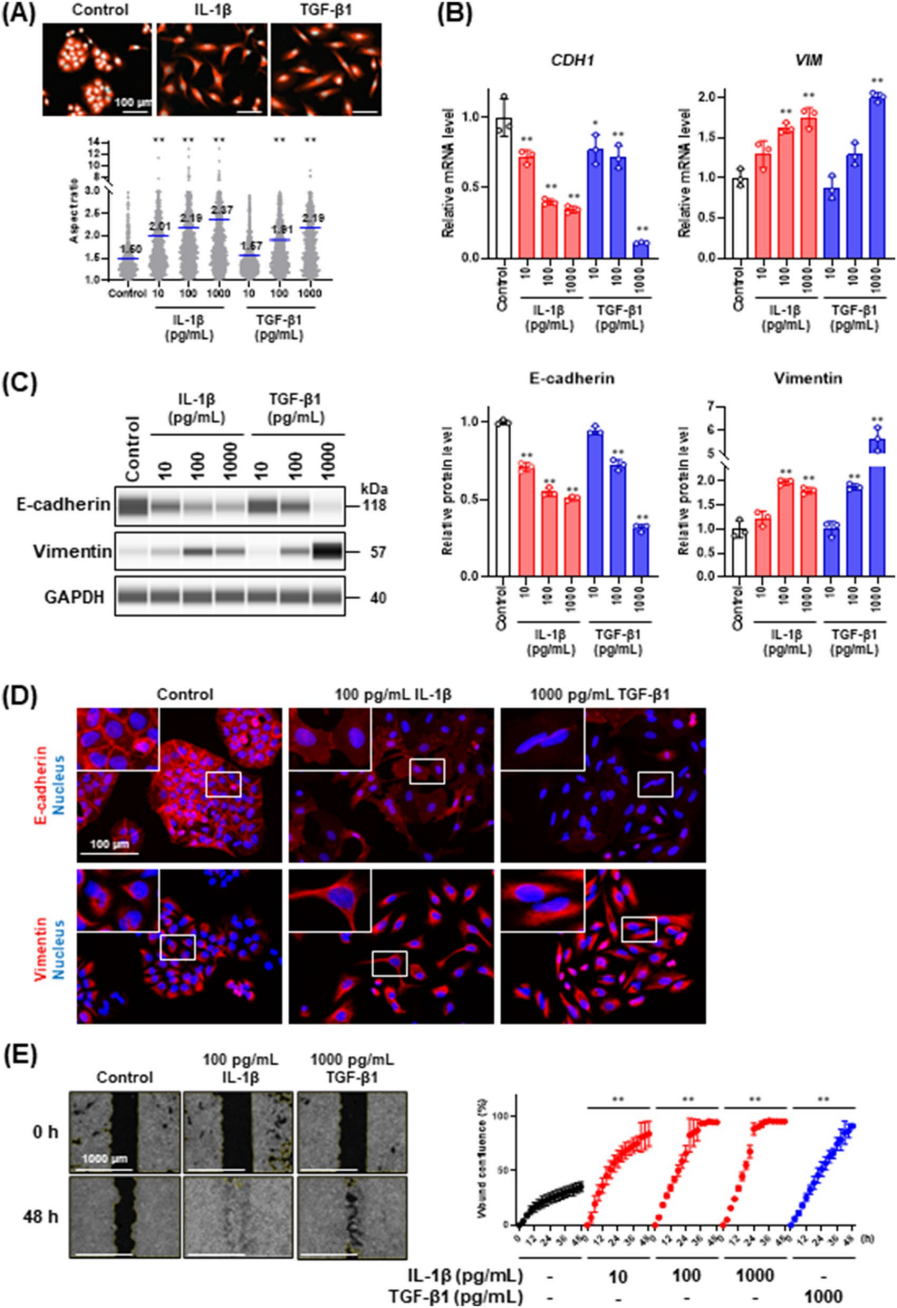
Induction of EMT in A549 cells stimulated with IL-1β or TGF-β1. **A** Quantitative analysis of morphological changes induced by IL-1β or TGF-β1 treatment. The cells were treated with IL-1β or TGF-β1 for 48 h and stained with Hoechst 33342 and CellTracker Red CMTPX dye. The cells were visualized under a CQ1 confocal quantitative image cytometer (upper panels). The aspect ratio (bottom panel) was analyzed as described in Materials and Methods. Values are means, *n* = 1000, one-way ANOVA followed by Tukey’s multiple comparison test. ** *P* < 0.01, compared with untreated control. **B** *CDH1* and *VIM* mRNA expression levels in cells treated with IL-1β or TGF-β1 for 48 h. Each mRNA expression level was normalized to the corresponding *ACTB* value and is presented as relative units to untreated control. Values are means ± SD, n = 3, one-way ANOVA followed by Tukey’s multiple comparison test. *P < 0.05, **P < 0.01, compared with untreated control. **C** Western blot analysis of E-cadherin and vimentin in cells treated with IL-1β or TGF-β1 for 48 h. Each protein level was normalized to the corresponding GAPDH value and is presented as relative units to untreated control (right panels). Values are means ± SD, n = 3, one-way ANOVA followed by Tukey’s multiple comparison test. **P < 0.01, compared with untreated control. **D** Immunofluorescence analysis of E-cadherin (upper panels) and vimentin (bottom panels) in cells treated with IL-1β or TGF-β1 for 48 h. Nuclei were stained with NucBlue. Fluorescence images were obtained by using a BZ-X710 fluorescence microscope. Insets show high-magnification images of the boxed areas. **E** Wound healing assay of cells treated with IL-1β or TGF-β1. Images (left panels) and wound confluence values (right panel) were obtained using a Lionheart FX automated live cell imager and Gen5 software, respectively. Values are means ± SD, n = 3, two-way ANOVA followed by Tukey’s multiple comparison test. **P < 0.01, compared with untreated control

As an initial experiment, the dose dependence of the EMT induction by IL-1β was investigated and compared with that by potent EMT inducer TGF-β1. To evaluate IL-1β-mediated EMT induction, E-cadherin and vimentin were selected as epithelial and mesenchymal markers, respectively, based on our previous study [25]. E-cadherin is an adhesion protein specifically located at the membrane of epithelial cells, whereas vimentin is an intermediate filament protein typically expressed in the cytoplasm of mesenchymal cells [1]. As shown in Fig. 1A, IL-1β dose-dependently induced morphological changes from a cobblestone-like shape to an elongated spindle shape by increasing the aspect ratio. Real-time PCR analysis (Fig. 1B) and western blot analysis (Fig. 1C) revealed that IL-1β downregulated E-cadherin (encoded by *CDH1*) and upregulated vimentin (encoded by *VIM*) in a dose-dependent manner. Interestingly, although EMT induction triggered by IL-1β occurred at a lower concentration than that by TGF-β1 (Fig. 1B and C), the exposure to IL-1β led to the co-expression of E-cadherin and vimentin, which is similar to the partial EMT phenotype described previously [32]. Consistently, immunofluorescence analysis showed that although the expression of E-cadherin was decreased and that of vimentin was increased after IL-1β treatment, the effect of IL-1β on these proteins was less than that of TGF-β1 (Fig. 1D). A full switch from an epithelial phenotype into a differentiated mesenchymal phenotype (complete EMT) was achieved by TGF-β1 treatment but not by IL-1β treatment, suggesting that the IL-1β-mediated phenotypic switch was a partial EMT. In addition, cell motility, one of the important characteristics of partial EMT, was more rapidly promoted by IL-1β than by TGF-β1 in the wound healing assay (Fig. 1E). Taken together, these results suggest that the treatment with IL-1β leads to the partial EMT-like phenotype with disruption of cell polarity and high migratory potential.

### IL-1β-mediated partial EMT is induced via EGFR phosphorylation

EMT in response to TGF-β1 is mediated predominantly via the Smad pathway [33]. Then, to investigate the involvement of the Smad pathway in the IL-1β-mediated partial EMT, we analyzed Smad2 and 3 phosphorylation levels in cells stimulated with IL-1β. As shown in Fig. S3, Smad2 and 3 were phosphorylated by TGF-β1 but not by IL-1β, indicating that the IL-1β-mediated partial EMT occurred independently of the Smad pathway.

As NF-κB p65 activation is an early event in IL-1β signaling [34], we investigated the involvement of NF-κB p65 in the IL-1β-mediated partial EMT. To determine whether NF-κB p65 induces the IL-1β-mediated partial EMT, cells were treated with the inhibitor of kappa-B α (IκBα) kinase inhibitor, BAY11-7082, for 4 h and then stimulated with IL-1β for an additional 48 h. Unfortunately, although the treatment of cells with BAY11-7082 led to the inhibition of NF-κB p65 phosphorylation (Fig. S4A), the IL-1β-triggered decrease of E-cadherin and increase of vimentin could not be repressed at both mRNA levels (Fig. S4B) and protein levels (Fig. S4C). These results suggest that the IL-1β-mediated partial EMT is induced independently of NF-κB p65 activation.

**Fig. 2:**
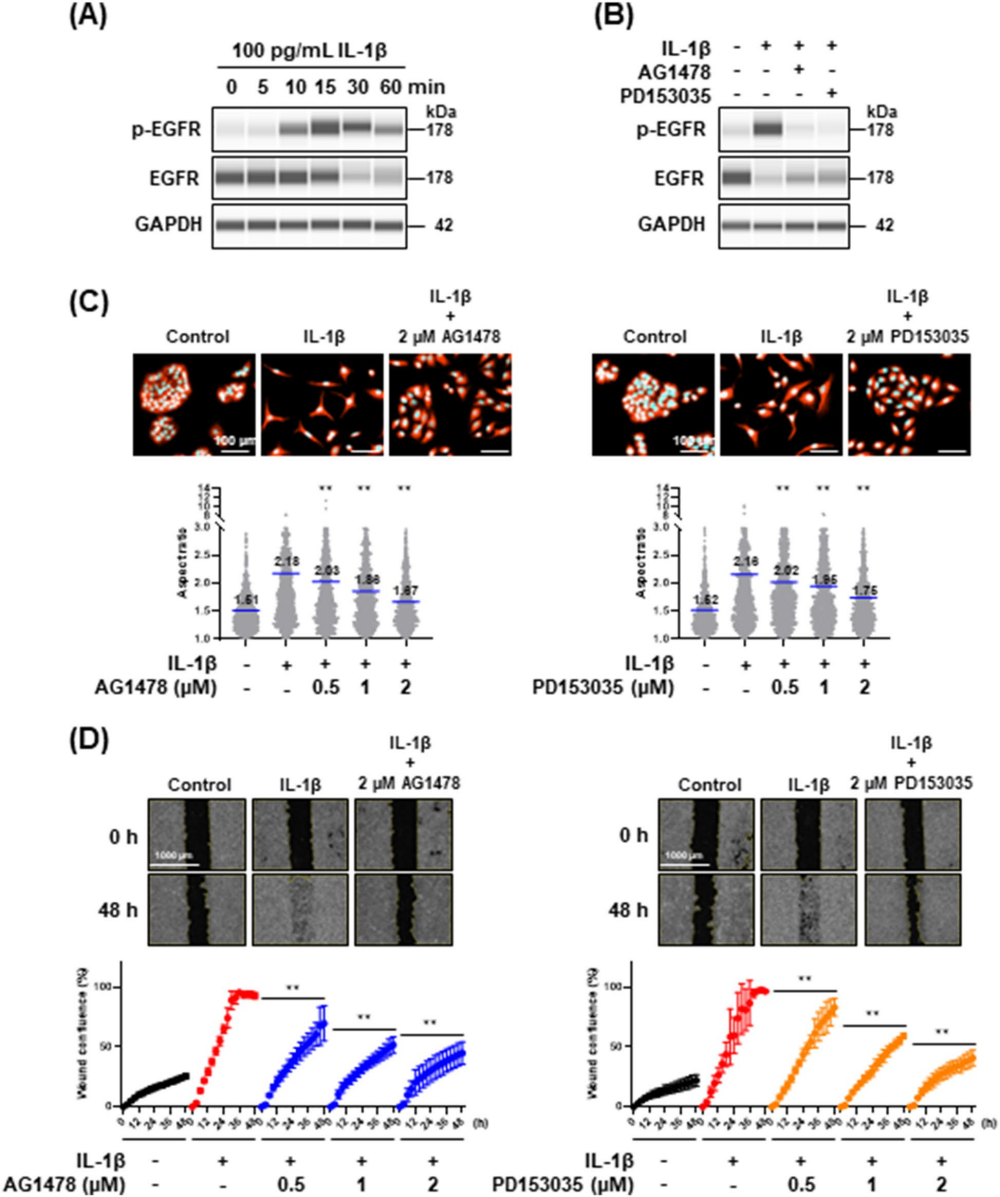
Effect of EGFR tyrosine kinase inhibitors (TKIs), AG1478 and PD153035, on the phenotypic properties of A549 cells stimulated with IL-1β. **A** Western blot analysis of EGFR phosphorylation in cells stimulated with 100 pg/mL IL-1β for the indicated times. GAPDH was used as internal control. **B** Effect of EGFR TKIs on the EGFR phosphorylation. The cells were treated with 2 μM EGFR TKIs for 4 h and then stimulated with 100 pg/mL IL-1β for an additional 30 min. GAPDH was used as internal control. **C** Quantitative analysis of morphological changes induced by IL-1β treatment. The cells were treated with EGFR TKIs for 4 h and then stimulated with 100 pg/mL IL-1β for an additional 48 h. The cells were stained with Hoechst 33342 and CellTracker Red CMTPX dye and then visualized under a CQ1 confocal quantitative image cytometer (upper panels). The aspect ratio (bottom panels) was analyzed as described in Materials and Methods. Values are means, *n* = 1000, one-way ANOVA followed by Tukey’s multiple comparison test. ** *P* < 0.01, compared with IL-1β-treated cells. **D** Wound healing assay of cells treated with 100 pg/mL IL-1β in the presence of EGFR TKIs. Images (upper panels) and wound confluence values (bottom panels) were obtained using a Lionheart FX automated live cell imager and Gen5 software, respectively. Values are means ± SD, n = 3, two-way ANOVA followed by Tukey’s multiple comparison test. **P < 0.01, compared with IL-1β-treated cells

IL-1β transactivates EGFR in pulmonary epithelial cells through its receptors [35]. Thus, we analyzed the phosphorylation levels of EGFR at Tyr1068 in IL-1β-stimulated cells and found a marked increase in EGFR phosphorylation levels after IL-1β stimulation (Fig. 2A). EGFR levels were downregulated in IL-1β-stimulated cells probably due to ligand-induced receptor internalization and lysosomal-mediated degradation [36]. To investigate the participation of the EGFR signaling pathway in the IL-1β-mediated partial EMT, we used two EGFR tyrosine kinase inhibitors (TKIs), AG1478 and PD153035. Both TKIs completely inhibited the IL-1β-induced EGFR phosphorylation (Fig. 2B) and dose-dependently inhibited morphological changes induced by IL-1β treatment (Fig. 2C). In addition, the wound healing assay revealed that the IL-1β-promoted cell motility was also decreased by the EGFR TKIs in a dose-dependent manner (Fig. 2D). These results indicate that the EGFR signaling pathway is involved in the disruption of cell polarity and the increase in migratory potential.

**Fig. 3:**
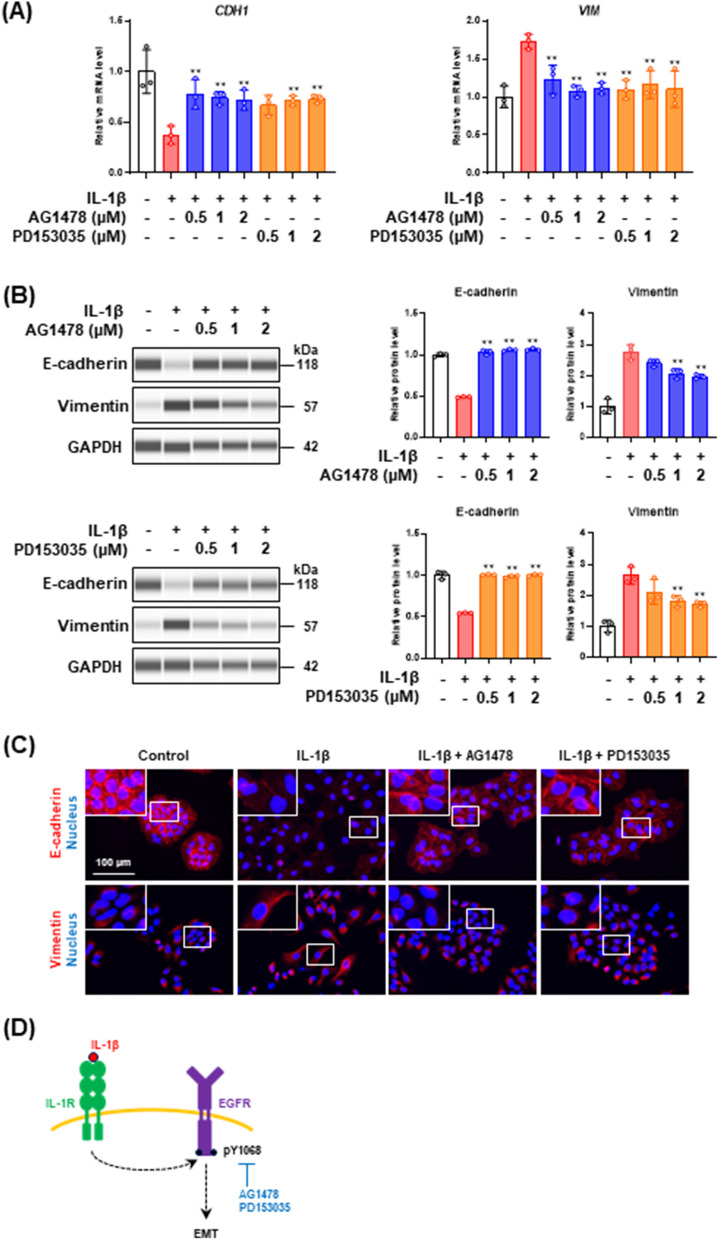
Effect of EGFR tyrosine kinase inhibitors (TKIs), AG1478 and PD153035, on EMT markers in A549 cells stimulated with IL-1β. **A** *CDH1* and *VIM* mRNA expression levels in cells treated with 100 pg/mL IL-1β in the presence of EGFR TKIs for 48 h. Each mRNA expression level was normalized to the corresponding *ACTB* value and is presented as relative units compared with untreated control. Values are means ± SD, n = 3, one-way ANOVA followed by Tukey’s multiple comparison test. **P < 0.01, compared with IL-1β-treated cells. **B** Western blot analysis of E-cadherin and vimentin in cells treated with 100 pg/mL IL-1β in the presence of EGFR TKIs for 48 h. Each protein level was normalized to the corresponding GAPDH value and is presented as relative units to untreated control. Values are means ± SD, n = 3, one-way ANOVA followed by Tukey’s multiple comparison test. **P < 0.01, compared with IL-1β-treated cells. **C** Immunofluorescence analysis of E-cadherin (upper panels) and vimentin (bottom panels) in cells treated with 100 pg/mL IL-1β in the presence of 2 μM EGFR TKIs for 48 h. Nuclei were stained with NucBlue. Fluorescence images were obtained by using a BZ-X710 fluorescence microscope. Insets show high-magnification images of the boxed areas. **D** A schematic model of the signaling pathways involved in IL-1β-mediated partial EMT induction

To further confirm whether the EGFR signaling pathway is committed to the IL-1β-mediated partial EMT, we analyzed the effects of the EGFR TKIs on the mRNA and protein levels of epithelial and mesenchymal markers. Real-time PCR analysis (Fig. 3A) and western blot analysis (Fig. 3B) indicated that the EGFR TKIs repressed IL-1β-induced decrease of E-cadherin and increase of vimentin, although the effective concentrations were different. Immunofluorescence staining revealed that E-cadherin localization was disrupted by IL-1β but retained by the treatment with EGFR TKIs (Fig. 3C). EGFR TKIs treatment repressed the filamentous structure of vimentin formed in the IL-1β-stimulated cells (Fig. 3C). These results indicate that the IL-1β-mediated partial EMT is induced via the transactivation of the EGFR signaling pathway (Fig. 3D).

### IL-1β-induced EGFR transactivation is triggered by MMP/ADAM-mediated shedding

**Fig. 4:**
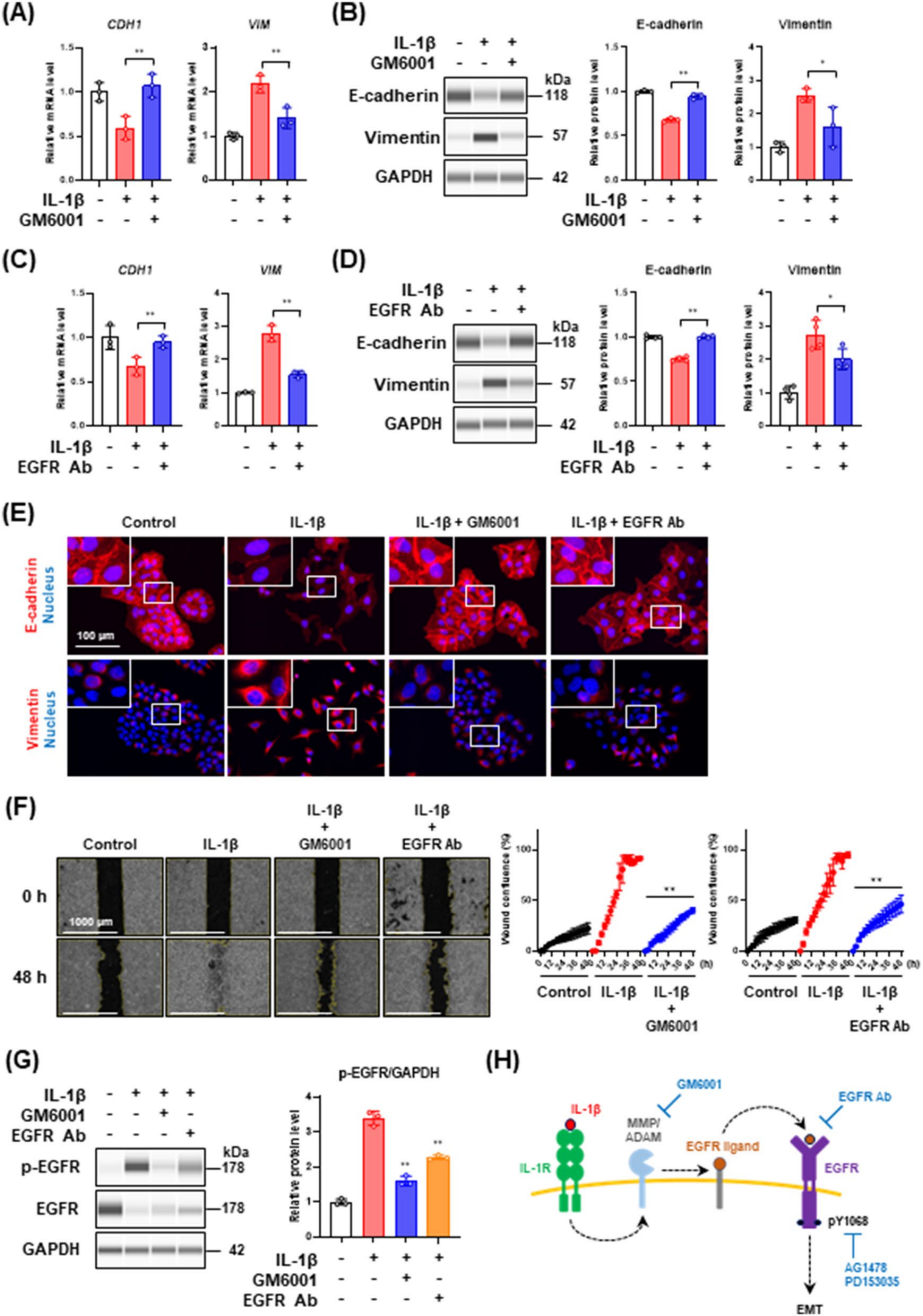
Crosstalk between EGFR transactivation and IL-1β-dependent EMT induction. **A** and** C** *CDH1* and *VIM* mRNA expression levels in cells treated with 100 pg/mL IL-1β in the presence of 50 μM GM6001 (**A**) or 10 μg/mL anti-EGFR Ab (**C**) for 48 h. Each mRNA expression level was normalized to the corresponding *ACTB* value and is presented as relative units to untreated control. Values are means ± SD, n = 3, one-way ANOVA followed by Tukey’s multiple comparison test. **P < 0.01, compared with IL-1β-treated cells. **B** and **D** Western blot analysis of E-cadherin and vimentin in cells treated with 100 pg/mL IL-1β in the presence of 50 μM GM6001 (**B**) or 10 μg/mL anti-EGFR Ab (**D**) for 48 h. Each protein level was normalized to the corresponding GAPDH value and is presented as relative to untreated control. Values are means ± SD, n = 4 or 3, one-way ANOVA followed by Tukey’s multiple comparison test. *P < 0.05, **P < 0.01, compared with IL-1β-treated cells. **E** Immunofluorescence analysis of E-cadherin (upper panels) and vimentin (bottom panels) in cells treated with 100 pg/mL IL-1β in the presence of 50 μM GM6001 or 10 μg/mL anti-EGFR Ab for 48 h. Nuclei were stained with NucBlue. Fluorescence images were obtained by using a BZ-X710 fluorescence microscope. Insets show high-magnification images of the boxed areas. **F** Wound healing assay of cells treated with 100 pg/mL IL-1β in the presence of 50 μM GM6001 or 10 μg/mL anti-EGFR Ab. Images (left panels) and wound confluence values (right panels) were obtained using a Lionheart FX automated live cell imager and Gen5 software, respectively. Values are means ± SD, n = 3, two-way ANOVA followed by Tukey’s multiple comparison test. **P < 0.01, compared with IL-1β-treated cells. **G** Western blot analysis of EGFR phosphorylation in cells stimulated with 100 pg/mL IL-1β in the presence of 50 μM GM6001 or 10 μg/mL anti-EGFR Ab for 30 min. EGFR phosphorylation level was normalized to the corresponding GAPDH value and is presented as relative units to untreated control. Values are means ± SD, n = 3, one-way ANOVA followed by Tukey’s multiple comparison test. **P < 0.01, compared with IL-1β-treated cells. **H** A schematic model of the signaling pathways involved in IL-1β-mediated partial EMT induction

It has been reported that the IL-1β-induced EGFR transactivation is mediated by matrix metalloproteinase (MMP)/a disintegrin and metalloproteinase (ADAM) activation [37]. In this pathway, the activated MMP/ADAM cleave and release various EGFR ligands, leading to subsequent EGFR transactivation [38]. Although MMP/ADAM inhibition is known to decrease IL-1β-induced cell motility [35, 37], the relationships between MMP/ADAM and partial EMT have not been reported. Then, to investigate the involvement of MMP/ADAM activation in both IL-1β-mediated EGFR transactivation and partial EMT induction, we treated the cells with the MMP/ADAM inhibitor, GM6001, and analyzed the expression of EMT markers. As shown in Fig. 4A and B, the treatment with GM6001 effectively suppressed the IL-1β-induced decrease of E-cadherin and increase of vimentin. Similarly, the treatment with the anti-EGFR antibody (Ab) to block the effect of EGFR ligands inhibited the decrease of E-cadherin and the increase of vimentin at both mRNA and protein levels (Fig. 4C and D). In addition, immunofluorescence analysis revealed that GM6001 and anti-EGFR Ab retained E-cadherin localized at cell–cell junctions that were disrupted by IL-1β stimulation (Fig. 4E, upper panels), and repressed filamentous vimentin formation by IL-1β stimulation (Fig. 4E, bottom panels). Furthermore, GM6001 and anti-EGFR Ab suppressed the IL-1β-induced increase in cell motility (Fig. 4F). To clarify whether GM6001 and neutralizing anti-EGFR Ab repressed the IL-1β-mediated EGFR transactivation, we treated the cells with GM6001 and anti-EGFR Ab and analyzed EGFR phosphorylation 30 min after IL-1β stimulation. As shown in Fig. 4G, both GM6001 and anti-EGFR Ab repressed the IL-1β-dependent phosphorylation of EGFR. Taken together, these results suggest that IL-1β-mediated EGFR transactivation and partial EMT induction are induced via MMP/ADAM activation to release soluble EGFR ligands (Fig. 4H).

### EGFR downstream PI3K/AKT signaling pathway contributes to IL-1β-mediated partial EMT

**Fig. 5:**
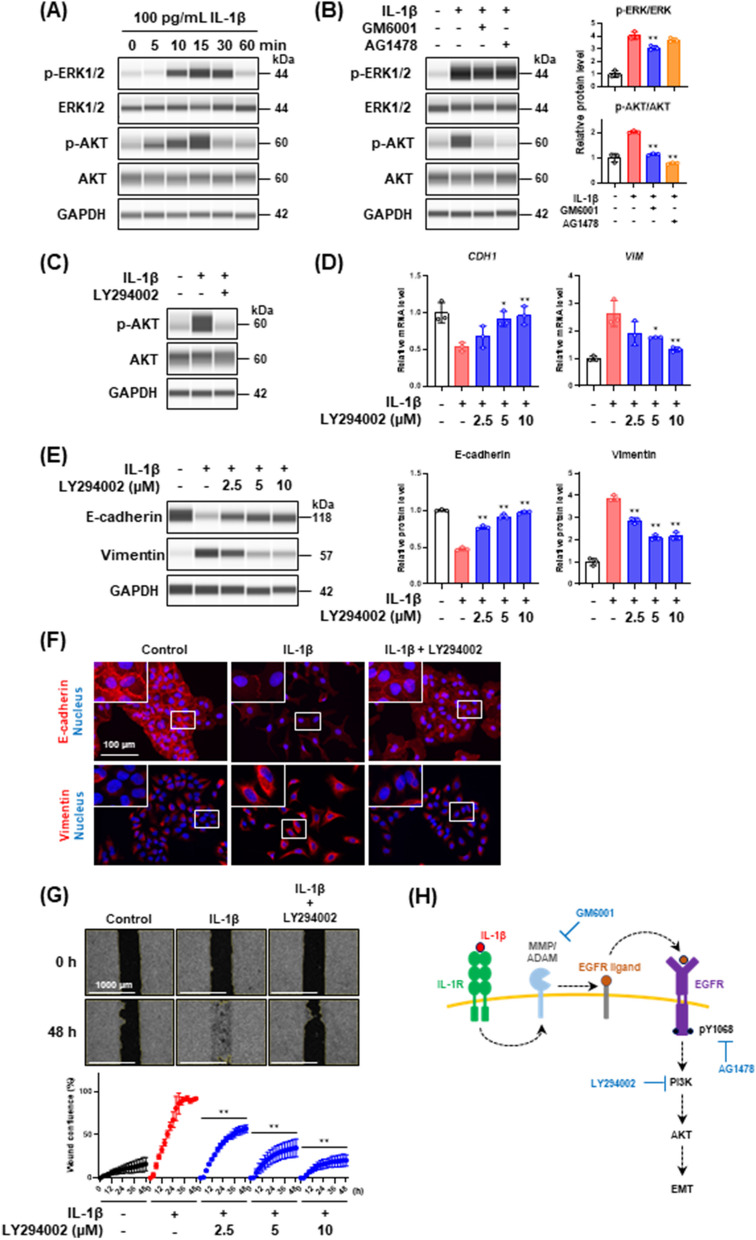
Downstream of EGFR signaling pathways transactivated by IL-1β. **A** Western blot analysis of ERK1/2 and AKT phosphorylation in cells stimulated with 100 pg/mL IL-1β for the indicated times. **B** Effect of GM6001 (50 μM) and AG1478 (2 μM) on ERK1/2 and AKT phosphorylation in cells stimulated with 100 pg/mL IL-1β for 15 min. Each phosphorylated protein level was normalized to the corresponding total protein level and is presented as relative units to untreated control. Values are means ± SD, n = 3, one-way ANOVA followed by Tukey’s multiple comparison test. **P < 0.01, compared with IL-1β-treated cells. **C** Western blot analysis of AKT phosphorylation in cells treated with 100 pg/mL IL-1β in the presence of 10 μM PI3K inhibitor, LY294002, for 15 min. **D** *CDH1* and *VIM* mRNA expression levels in cells treated with 100 pg/mL IL-1β in the presence of LY294002 for 48 h. Each mRNA expression level was normalized to the corresponding *ACTB* value and is presented as relative units to untreated control. Values are means ± SD, n = 3, one-way ANOVA followed by Tukey’s multiple comparison test. *P < 0.05, **P < 0.01, compared with IL-1β-treated cells. **E** Western blot analysis of E-cadherin and vimentin in cells treated with 100 pg/mL IL-1β in the presence of LY294002 for 48 h. Each protein level was normalized to the corresponding GAPDH value and is presented as relative units to untreated control. Values are means ± SD, n = 3, one-way ANOVA followed by Tukey’s multiple comparison test. **P < 0.01, compared with IL-1β-treated cells. **F** Immunofluorescence analysis of E-cadherin (upper panels) and vimentin (bottom panels) in cells treated with 100 pg/mL IL-1β in the presence of 10 μM LY294002 for 48 h. Nuclei were stained with NucBlue. Fluorescence images were obtained by using a BZ-X710 fluorescence microscope. Insets show high-magnification images of the boxed areas. **G** Wound healing assay of cells treated with 100 pg/mL IL-1β in the presence of LY294002. Images (upper panels) and wound confluence values (bottom panel) were obtained using a Lionheart FX automated live cell imager and Gen5 software, respectively. Values are means ± SD, n = 3, two-way ANOVA followed by Tukey’s multiple comparison test. **P < 0.01, compared with IL-1β-treated cells. **H** A schematic model of the signaling pathways involved in IL-1β-mediated partial EMT induction

Two key downstream signaling pathways of EGFR are the MEK/ERK and PI3K/AKT pathways [39]. First, we assessed the dynamics of ERK1/2 and AKT phosphorylation following stimulation with IL-1β. As shown in Fig. 5A, both ERK1/2 and AKT were rapidly phosphorylated by the IL-1β stimulation. Then, to clarify which pathway is downstream of the IL-1β-transactivated EGFR signaling pathway, we treated the cells with IL-1β in the presence of GM6001 (MMP/ADAM inhibitor) or AG1478 (EGFR TKI) and analyzed ERK1/2 and AKT phosphorylation levels. Interestingly, GM6001 and AG1478 almost completely inhibited AKT phosphorylation induced by IL-1β, but not ERK1/2 phosphorylation (Fig. 5B), indicating that PI3K/AKT is downstream of the IL-1β-transactivated EGFR signaling pathway.

We investigated the effects of PI3K inhibitor LY294002 to confirm whether the PI3K/AKT signaling pathway contributes to the IL-1β-mediated partial EMT. LY294002 completely inhibited the IL-1β-induced AKT phosphorylation (Fig. 5C) and dose-dependently inhibited the decrease of *CDH1* and the increase of *VIM* owing to IL-1β stimulation in real-time PCR analysis (Fig. 5D). Western blotting and immunofluorescence analysis also revealed that LY294002 repressed the alteration of EMT markers on the protein level and their localization (Fig. 5E and F). In addition, we found that the increased migration capability of IL-1β-stimulated cells was reduced by the treatment with LY294002 (Fig. 5G). These results indicate that the PI3K/AKT signaling pathway plays a key role in the IL-1β-mediated partial EMT induction (Fig. 5H).

### EGF or AREG stimulation is not sufficient to induce partial EMT

**Fig. 6:**
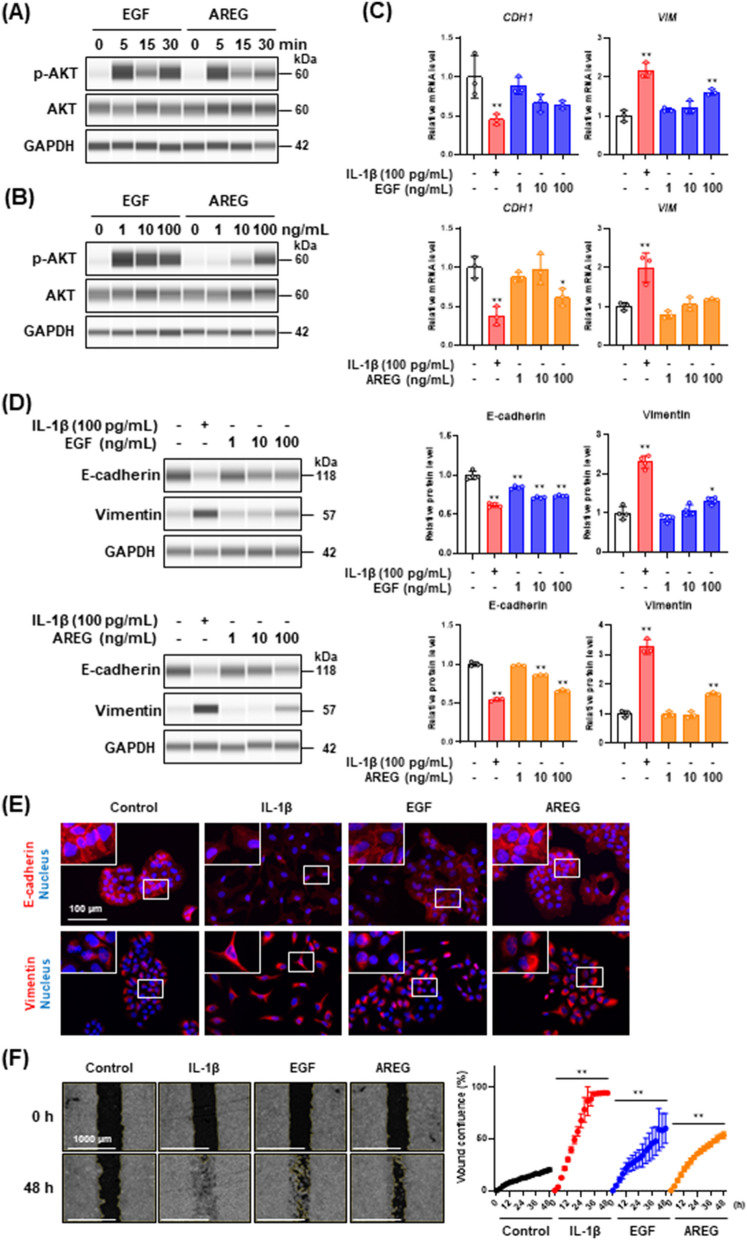
Effect of EGFR ligands, EGF and AREG, on the induction of EMT in A549 cells. **A** Western blot analysis of AKT phosphorylation in cells stimulated with EGF (100 ng/mL) or AREG (100 ng/mL) for the indicated times. **B** Western blot analysis of AKT phosphorylation in cells treated with different concentrations of EGF and AREG for 5 min. GAPDH was used as internal control. **C** *CDH1* and *VIM* mRNA expression levels in cells treated with 100 pg/mL IL-1β, EGF (upper panels), or AREG (bottom panels) for 48 h. Each mRNA expression level was normalized to the corresponding *ACTB* value and is presented as relative units to untreated control. Values are means ± SD, n = 3, one-way ANOVA followed by Tukey’s multiple comparison test. *P < 0.05, **P < 0.01, compared with untreated control. **D** Western blot analysis of E-cadherin and vimentin in cells treated with 100 pg/mL IL-1β, EGF (upper panels), or AREG (bottom panels) for 48 h. Each protein level was normalized to the corresponding GAPDH value and is presented as relative units to untreated control. Values are means ± SD, n = 3, one-way ANOVA followed by Tukey’s multiple comparison test. *P < 0.05, **P < 0.01, compared with untreated control. **E** Immunofluorescence analysis of E-cadherin (upper panels) and vimentin (bottom panels) in cells treated with 100 pg/mL IL-1β, 100 ng/mL EGF, or 100 ng/mL AREG for 48 h. Nuclei were stained with NucBlue. Fluorescence images were obtained by using a BZ-X710 fluorescence microscope. Insets show high-magnification images of the boxed areas. **F** Wound healing assay of cells treated with 100 pg/mL IL-1β, 100 ng/mL EGF, or 100 ng/mL AREG. Images (left panels) and wound confluence values (right panel) were obtained using a Lionheart FX automated live cell imager and Gen5 software, respectively. Values are means ± SD, n = 3, two-way ANOVA followed by Tukey’s multiple comparison test. **P < 0.01, compared with untreated control

As the above-mentioned results led us to speculate that an EGFR ligand could induce partial EMT in A549 cells, we treated the cells with an EGFR ligand and assessed EMT induction. In addition to the most widely known EGFR ligand, EGF, we investigated the EMT inductive effects of amphiregulin (AREG), which is highly expressed in this cell line [40]. The treatment with EGF and AREG rapidly induced AKT phosphorylation although the effective concentrations were different (Fig. 6A and B). Treatment of the cells with EGF and AREG dose-dependently decreased E-cadherin and increased vimentin at both mRNA and protein levels (Fig. 6C and D). However, changes in the expression of these EMT markers caused by the EGF and AREG treatment were less marked than that by IL-1β treatment even at higher concentrations. Consistent with these observations, immunofluorescence analysis revealed that although E-cadherin expression was decreased by the EGF and AREG treatment, its localization on cell–cell junctions remained unchanged (Fig. 6E, upper panels). Furthermore, clear filamentous structures of vimentin were not formed by the treatment with these EGFR ligands (Fig. 6E, bottom panels). Wound healing assay revealed that EGF and AREG little affected cell motility compared with IL-1β (Fig. 6F). Together, the results suggest that the treatment with EGFR ligands, even at higher concentrations, is insufficient for the induction of partial EMT as observed in IL-1β-stimulated cells.

### EGFR-independent MEK/ERK signaling pathway contributes to IL-1β-mediated partial EMT

**Fig. 7:**
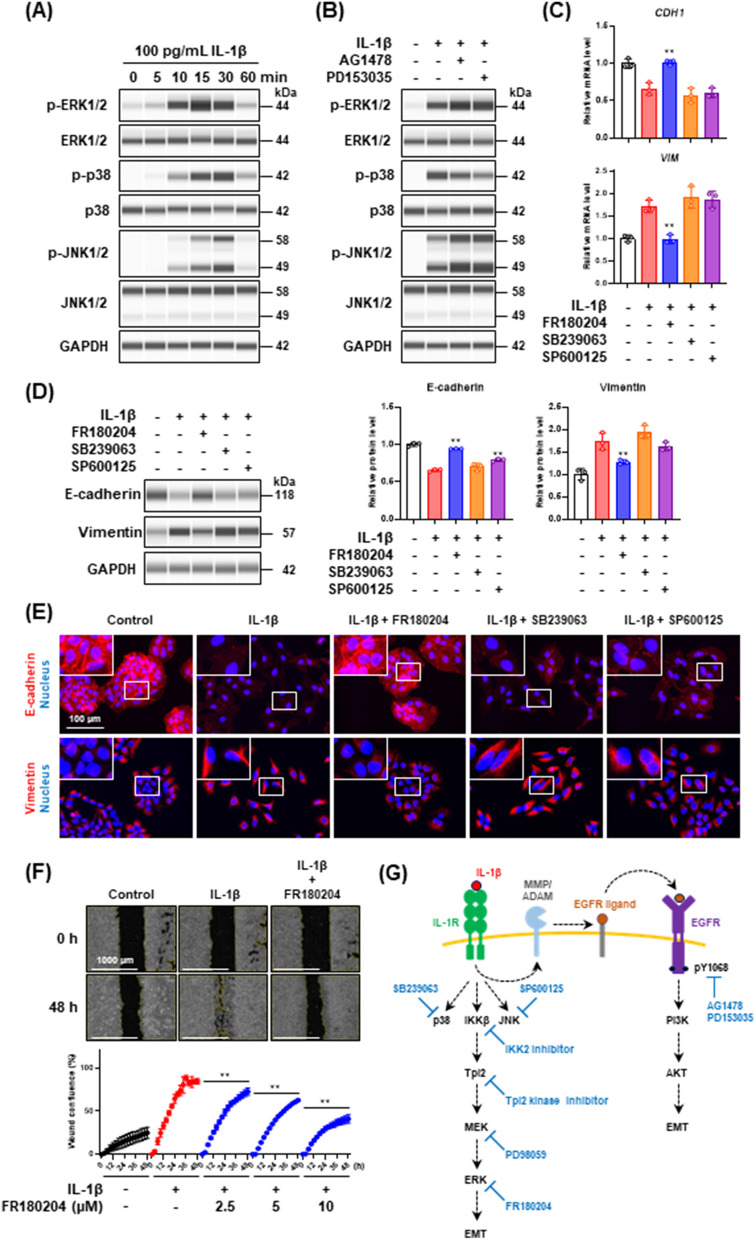
Identification of MAPK involved in IL-1β-dependent EMT induction. **A** Western blot analysis of ERK1/2, p38, and JNK1/2 phosphorylation in cells stimulated with IL-1β for the indicated times. **B** Effect of EGFR TKIs (2 μM) on ERK1/2, p38, and JNK1/2 phosphorylation in cells stimulated with 100 pg/mL IL-1β for 30 min. **C** *CDH1* and *VIM* mRNA expression levels in cells treated with IL-1β in the presence of MAPK inhibitors, FR180204, SB239063, or SP600125, for 48 h. Each mRNA expression level was normalized to the corresponding *ACTB* value and is presented as relative units to untreated control. Values are means ± SD, n = 3, one-way ANOVA followed by Tukey’s multiple comparison test. **P < 0.01, compared with IL-1β-treated cells. **D** Western blot analysis of E-cadherin and vimentin in cells treated with IL-1β in the presence of MAPK inhibitors for 48 h. Each protein level was normalized to the corresponding GAPDH value and is presented as relative units to untreated control. Values are means ± SD, n = 3, one-way ANOVA followed by Tukey’s multiple comparison test. **P < 0.01, compared with IL-1β-treated cells. **E** Immunofluorescence analysis of E-cadherin (upper panels) and vimentin (bottom panels) in cells treated with IL-1β in the presence of MAPK inhibitors for 48 h. Nuclei were stained with NucBlue. Fluorescence images were obtained by using a BZ-X710 fluorescence microscope. Insets show high-magnification images of the boxed areas. **F** Wound healing assay of cells treated with IL-1β in the presence of ERK inhibitor, FR180204. Images (upper panels) and wound confluence values (bottom panel) were obtained using a Lionheart FX automated live cell imager and Gen5 software, respectively. Values are means ± SD, n = 3, two-way ANOVA followed by Tukey’s multiple comparison test. **P < 0.01, compared with IL-1β-treated cells. **G** A schematic model of the signaling pathways involved in IL-1β-mediated partial EMT induction

On the basis of the above findings, we hypothesized that besides the EGFR-dependent PI3K/AKT signaling pathway, an EGFR-independent signaling pathway may be required for the induction of IL-1β-mediated partial EMT. It has been reported that IL-1β activates mitogen-activated protein kinase (MAPK) signaling pathways, including the ERK, p38, and c-jun N-terminal kinase (JNK) pathways [41]. We noted marked increases in the phosphorylation levels of ERK1/2, p38, and JNK1/2 in IL-1β-stimulated cells, as reported previously (Fig. 7A), which were induced in an EGFR-independent manner because EGFR TKIs (AG1478 and PD153035) did not completely repress these phosphorylation levels (Fig. 7B). To investigate the contribution of MAPK signaling pathways to the IL-1β-mediated partial EMT induction, we examined the effects of MAPK inhibitors on the expression of EMT markers in cells stimulated with IL-1β. FR180204, SB239063, and SP600125 were used as ERK1/2, p38, and JNK1/2 inhibitors, respectively. As shown in Fig. 7C and D, the ERK1/2 inhibitor FR180204 inhibited IL-1β-induced E-cadherin downregulation and vimentin upregulation at both mRNA (Fig. 7C) and protein (Fig. 7D) levels. Immunofluorescence analysis also revealed that FR180204 repressed the IL-1β-mediated E-cadherin disruption and vimentin formation (Fig. 7E). On the other hand, clear inhibitory effects were not observed by the treatment with p38 inhibitor SB239063 and JNK1/2 inhibitor SP600125 (Fig. 7C–E). The cell motility increased by IL-1β was also repressed by treating the cells with FR180204 in a dose-dependent manner (Fig. 7F). These results suggest that the ERK signaling pathway that is downstream of IL-1R is required for the induction of IL-1β-mediated partial EMT.

At present, ERK1/2 are the only known physiological substrates of MEK1/2 [42, 43]. To further confirm whether the ERK signaling pathway contributes to the IL-1β-mediated partial EMT induction, we assessed the effect of MEK inhibitor PD98059 on the cells stimulated with IL-1β. PD98059 completely abolished ERK1/2 phosphorylation (Fig. S5A) and inhibited the decrease of E-cadherin and the increase of vimentin at both mRNA and protein levels in IL-1β-stimulated cells (Fig. S5B and C). The inhibitory effects of PD98059 on the IL-1β-mediated partial EMT induction were also confirmed by immunofluorescence analysis (Fig. S5D). Furthermore, PD98059 dose-dependently repressed the IL-1β-induced increase in cell motility (Fig. S5E). These results suggest that the MEK/ERK pathway plays a key role in the induction of the IL-1β-mediated partial EMT phenotype. In addition, the phospho-kinase assay revealed that both cytoplasmic and nuclear targets of ERK1/2, such as cAMP response element binding protein (CREB), c-jun, p90 ribosomal S6 kinase (RSK), and mitogen and stress-activated kinase (MSK), were phosphorylated by IL-1β stimulation (Fig S6A and B), indicating that the MEK/ERK pathway is activated during the induction of IL-1β-mediated partial EMT.

In response to inflammatory stimulation, the IκB kinase (IKK) complex phosphorylates NF-κB1 p105, which results in the proteolytic degradation of p105 and the subsequent release of tumor progression locus 2 (Tpl2). The direct phosphorylation and activation of MEK by Tpl2 is followed by the phosphorylation and activation of ERK [44, 45]. To obtain deeper insight into how the upstream of the MEK/ERK signaling pathway is involved in the IL-1β-mediated partial EMT induction, we treated the cells with IKKβ or Tpl2 kinase inhibitors and assessed EMT marker expressions. Treatment of cells with IKKβ or Tpl2 kinase inhibitors attenuated the MEK1/2 and ERK1/2 phosphorylation induced by IL-1β stimulation (Fig. S7A), indicating that MEK1/2 and ERK1/2 are activated downstream of IKKβ and Tpl2 kinase. Real-time PCR analysis (Fig. S7B) and western blot analysis (Fig. S7C) established that both inhibitors repressed the decrease of E-cadherin and the increase of vimentin, which were induced by IL-1β. These effects were also confirmed by immunofluorescence analysis (Fig. S7D). Taken together, the results suggest that MEK/ERK signaling pathway activated by IKKβ and Tpl2 kinase plays a key role in the IL-1β-mediated partial EMT induction (Fig. 7G).

### PI3K/AKT and MEK/ERK signaling pathways are required for IL-1β-mediated partial EMT induction

**Fig. 8:**
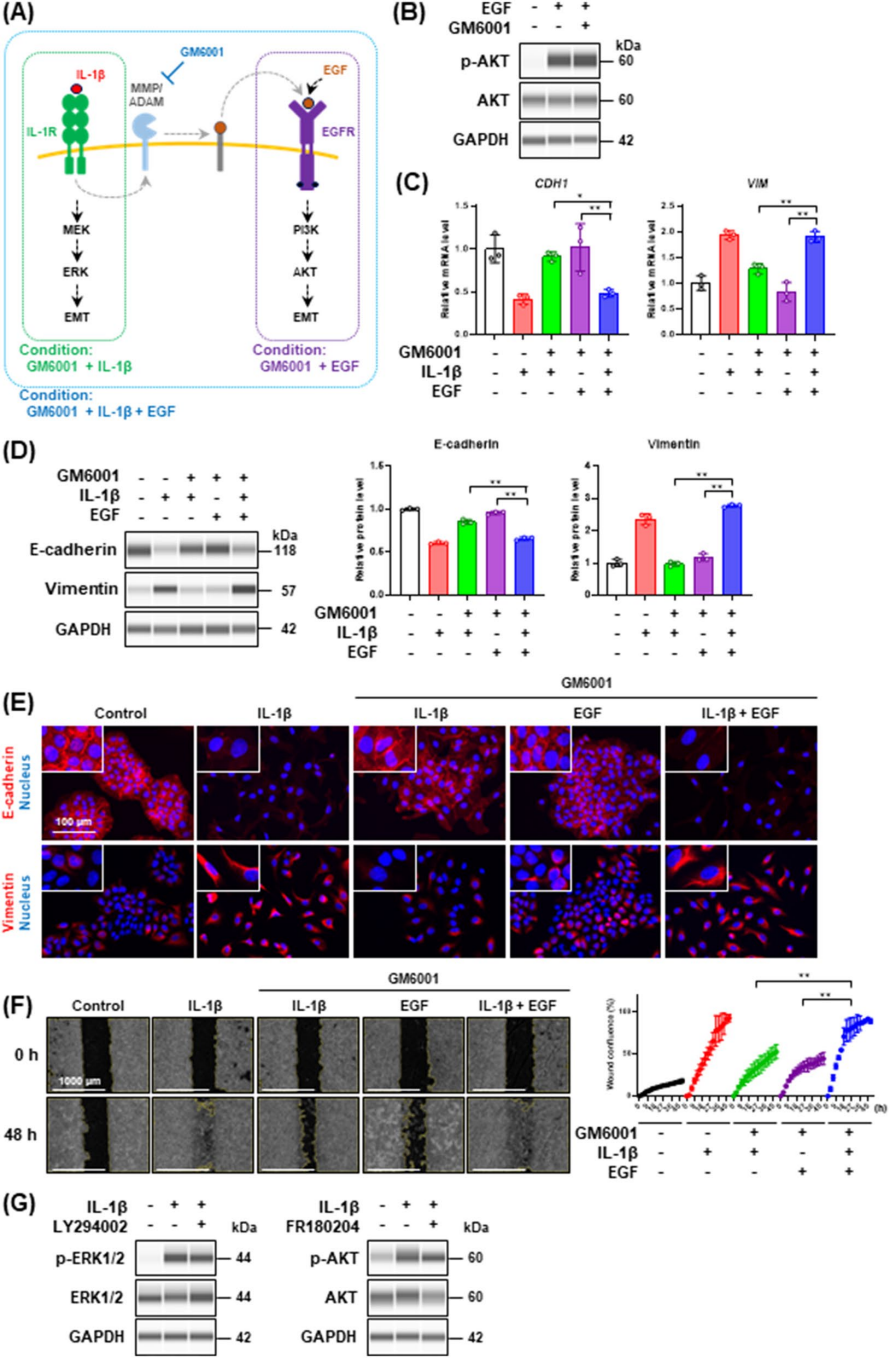
Crosstalk between MEK/ERK pathway and PI3K/AKT pathway in IL-1β-dependent EMT induction. **A** Schematic of the experimental setup. **B** Western blot analysis of AKT phosphorylation in cells treated with 1 ng/mL EGF in the presence of 50 μM GM6001 for 5 min. **C** *CDH1* and *VIM* mRNA expression levels in cells treated with IL-1β and/or EGF in the presence of GM6001 for 48 h. Each mRNA expression level was normalized to the corresponding *ACTB* value and is presented as relative units to untreated control. Values are means ± SD, n = 3, one-way ANOVA followed by Tukey’s multiple comparison test. *P < 0.05, **P < 0.01. **D** Western blot analysis of E-cadherin and vimentin in cells treated with IL-1β and/or EGF in the presence of GM6001 for 48 h. Each protein level was normalized to the corresponding GAPDH value and is presented as relative units to untreated control. Values are means ± SD, n = 3, one-way ANOVA followed by Tukey’s multiple comparison test. **P < 0.01. **E** Immunofluorescence analysis of E-cadherin (upper panels) and vimentin (bottom panels) in cells treated with IL-1β and/or EGF in the presence of GM6001 for 48 h. Nuclei were stained with NucBlue. Fluorescence images were obtained by using a BZ-X710 fluorescence microscope. Insets show high-magnification images of the boxed areas. **F** Wound healing assay of cells treated with IL-1β and/or EGF in the presence of GM6001. Images (left panels) and wound confluence values (right panel) were obtained using a Lionheart FX automated live cell imager and Gen5 software, respectively. Values are means ± SD, n = 3, two-way ANOVA followed by Tukey’s multiple comparison test. **P < 0.01. **G** Western blot analysis of ERK1/2 and AKT phosphorylation in cells stimulated with IL-1β in the presence of AKT inhibitor (LY294002) and ERK inhibitor (FR180204), respectively

The aforementioned results suggested that the activation of both PI3K/AKT and MEK/ERK pathways is required for the IL-1β-mediated partial EMT induction, and the activation of either pathway is not sufficient. To clarify this, we performed the following experiments using MMP/ADAM inhibitor GM6001. As mentioned above, GM6001 inhibited the MMP/ADAM-mediated EGFR transactivation and the EGFR-mediated PI3K/AKT pathway but not the IL-1R-mediated MEK/ERK pathway (Figs. 4G, 5B, and 8A, green dotted box). On the other hand, EGF activated the EGFR-mediated PI3K/AKT pathway even in the presence of GM6001 (Fig. 8B), but not the IL-1R-mediated MEK/ERK pathway (Fig. 8A, purple dotted box). From these observations, we hypothesized that if the IL-1β-mediated partial EMT were triggered by the activation of both EGFR-mediated PI3K/AKT and IL-1R-mediated MEK/ERK pathways, the IL-1β-mediated partial EMT would be induced by the co-treatment of the cells with IL-1β and EGF even in the presence of GM6001 (Fig. 8A, blue dotted box).

As shown in Fig. 8C and D, co-treatment of the cells with IL-1β and EGF induced the decrease of E-cadherin and the increase of vimentin at both mRNA and protein levels even in the presence of GM6001. Immunofluorescence analysis also revealed that the suppressive effect of GM6001 on the induction of IL-1β-mediated partial EMT was abrogated in the presence of both IL-1β and EGF (Fig. 8E). In addition, the IL-1β-promoted cell motility was repressed by the treatment with GM6001, but the repression was canceled by the co-treatment with IL-1β and EGF (Fig. 8F). These results suggest that the induction of IL-1β-mediated partial EMT is activated by the addition of EGFR ligands even though it is repressed by GM6001 owing to the inactivation of the EGFR-dependent PI3K/AKT signaling pathway. Furthermore, the treatment with LY294002 (PI3K inhibitor) or FR180204 (ERK1/2 inhibitor) did not completely repress ERK1/2 or AKT phosphorylation (Fig. 8G), respectively, indicating that the activation of the PI3K/AKT and MEK/ERK signaling pathways by IL-1β simultaneously induced and coordinately promoted partial EMT induction.

## Discussion

**Fig. 9:**
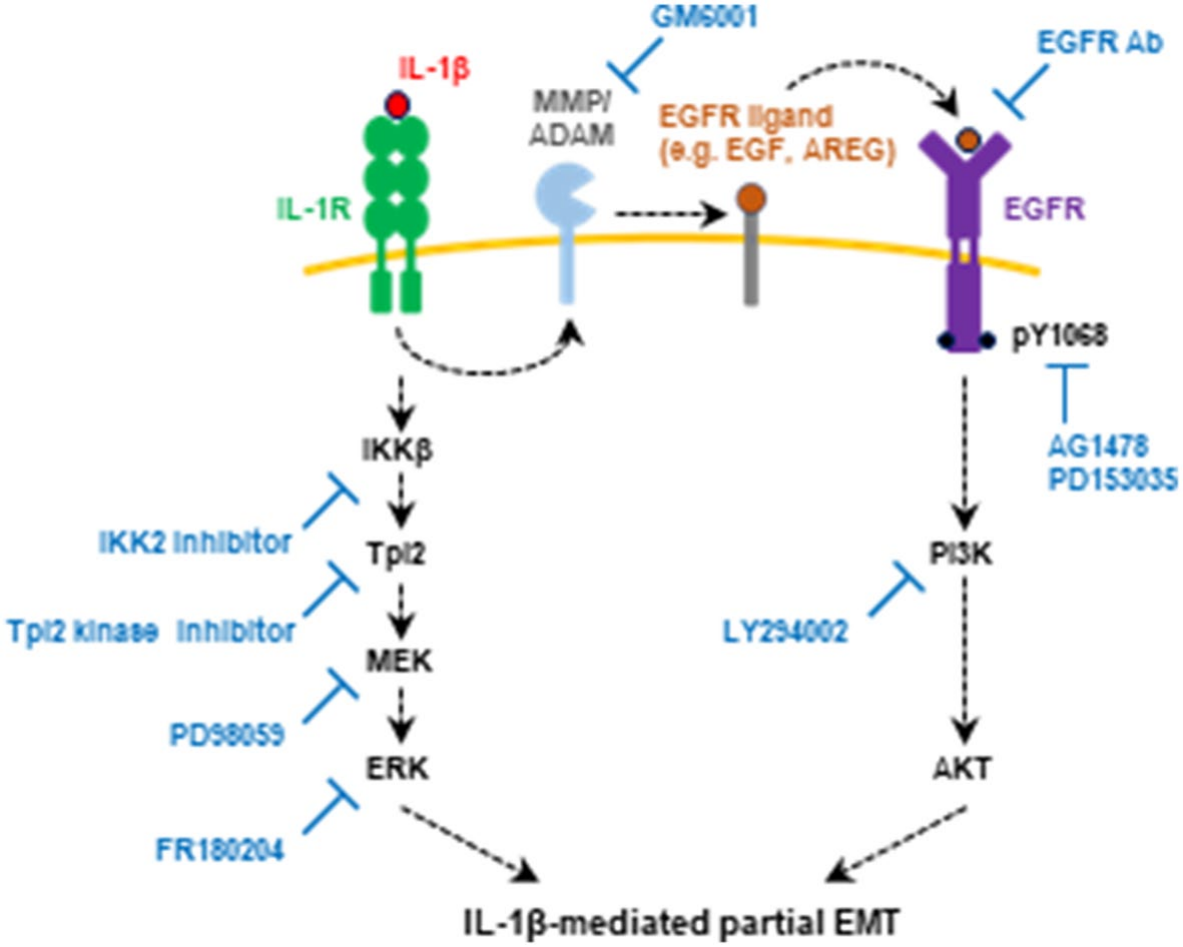
Proposed mechanism underlying IL-1β-mediated partial EMT induction. IL-1β induces EGFR transactivation in an MMP/ADAM-dependent apparatus and then activates EGFR downstream of the PI3K/AKT signaling pathway. In parallel, IL-1β activates the MEK/ERK signaling pathway via IKKβ/Tpl2-mediated phosphorylation. As a result, PI3K/AKT and MEK/ERK signaling pathways coordinately induce IL-1β-mediated partial EMT. Arrows show the effects triggered by IL-1β in the absence of the indicated inhibitors. The inhibitors are shown at the respective positions where they exert their inhibitory effects in the signaling pathways

EMT is a cell plasticity process that mediates embryonic development, tissue repair, and disease progression including organ fibrosis and cancer metastasis. It is induced by the activation of multiple signaling pathways such as TGF-β, Notch, and Wnt-mediated signaling pathways [46]. We and other researchers have independently reported EMT induction by proinflammatory cytokine IL-1β in various cell types [25–28]. IL-1β is one of the key mediators of inflammatory response, but the downstream signaling mechanism for EMT induction is not well understood. In this study, we found that two independent signaling pathways, EGFR-dependent PI3K/AKT and IL-1R-dependent MEK/ERK signaling pathways, are required for the induction of IL-1β-mediated EMT (Fig. 9).

Initially, we found that IL-1β stimulation leads to the partial EMT-like phenotype, including the gain of mesenchymal marker (vimentin) and higher migratory potential, without the complete loss of epithelial marker (E-cadherin) (Fig. 1). Other factors initiating partial EMT have been identified as well, including TGF-β [47, 48], hypoxia [49], and leptin [50]. However, there are no reports of the relationship between IL-1β and partial EMT induction. Partial EMT plays a critical role in tumor aggressiveness, invasion, migration, and metastasis along with therapeutic resistance [15]. Therefore, a concise understanding of the molecular mechanisms underlying partial EMT is clinically important for both the diagnosis and treatment of cancer. To the best of our knowledge, this is the first report that describes the signaling pathways underlying the IL-1β-mediated partial EMT induction.

EGFR transactivation is triggered by multiple receptor tyrosine kinases (RTKs), G-protein coupled receptors (GPCRs), cytokine receptors, and integrins [51, 52]. It is also involved in such physiological processes as DNA synthesis, cell cycle progression, and cell migration [53]. On the other hand, aberrant EGFR activation has been implicated in several human diseases, including pulmonary fibrosis, cancer progression, cardiovascular diseases, and Alzheimer’s disease [54]. In addition, EGFR mutations aberrantly activate the PI3K/AKT and MEK/ERK pathway, playing an important role in drug resistance and progression of NSCLC [39, 55]. EGFR transactivation is considered to be evoked by ligand-dependent and ligand-independent pathways. In the ligand-dependent pathways, MMP/ADAM-mediated ectodomain shedding of ligands, such as EGF and AREG, is required to release soluble ligands into extracellular space, and the released ligands activate EGFR within both autocrine and paracrine pathways [56]. On the other hand, the ligand-independent pathways involve the activation of intracellular protein tyrosine kinases such as Src family proteins that mediate the phosphorylation of EGFR in its cytosolic domain [57]. Both ligand-dependent and ligand-independent pathways have been suggested in IL-1β-mediated EGFR transactivation [35, 37]. Here, we showed that in the induction of IL-1β-mediated partial EMT, EGFR transactivation is mediated by ligand-dependent pathways because the IL-1β-mediated EGFR phosphorylation is repressed by MMP/ADAM inhibitor GM6001 and neutralizing anti-EGFR Ab (Fig. 4G), but not by Src inhibitor PP1 (Fig. S8).

Two primary downstream signaling pathways of EGFR are the PI3K/AKT and MEK/ERK pathways [39]. Previously, Matsuo et al. showed that the ADAM-mediated EGFR transactivation stimulated by fibronectin induced the activation of both AKT and ERK pathways that were inhibited by MMP/ADAM inhibitor GM6001 [58]. On the other hand, in this study, we found that the MMP/ADAM-mediated EGFR transactivation stimulated by IL-1β induced the activation of the PI3K/AKT pathway but not the MEK/ERK pathway, because AKT phosphorylation was significantly inhibited by MMP/ADAM inhibitor GM6001 and EGFR TKI AG1478 (Fig. 5B). The reason behind this discrepancy likely lies the difference in the activation processes of ERK pathway. Our study suggests that IL-1β-mediated phosphorylation of AKT is activated downstream of EGFR, whereas that of ERK1/2 is mainly activated downstream of IL-1R signaling cascade (Fig. 7G). In addition, changes of EMT markers triggered by IL-1β stimulation were repressed by the treatment with PI3K inhibitor LY294002, indicating that the PI3K/AKT signaling pathway plays a significant role in the IL-1β-mediated partial EMT induction (Fig. 5H). The PI3K/AKT pathway is involved in the regulation of various cell processes such as cell proliferation, apoptosis, and motility [59]. Accumulating evidence has revealed that the PI3K/AKT pathway is a key signaling mediator for EMT induction by upregulating EMT-related transcriptional factors that repress epithelial marker expression and promote mesenchymal marker expression [60, 61]. For example, NF-κB activation by PI3K/AKT induces the expression of Snail, an EMT-related transcriptional factor, causing EMT progression and metastasis development [20]. It was also reported that TNF-α-induced EMT is mediated by the stabilization of Snail through the activation of the PI3K/AKT/glycogen synthase kinase-3β pathway [62]. In addition, Chang et al. showed that the activation of the PI3K/AKT/mammalian target of rapamycin pathway is required for the EMT induction in prostate cancer cells [63]. Most recently, Chen et al. revealed that IL-1β promote tumor growth and metastasis by inducing the EMT through the activation of the PI3K/AKT/FOXO3A pathway [64]. Thus, it may be reasonable to assume that the activation of PI3K/AKT is crucial for the induction of IL-1β-mediated partial EMT.

Our results revealed that the activation of the PI3K/AKT pathway via EGFR transactivation is necessary, but not sufficient, for the induction of IL-1β-mediated partial EMT, because the treatment with EGFR ligands (EGF and AREG) is not adequate to induce the partial EMT-like phenotype (Fig. 6). The effect of EGF on the induction of EMT in A549 cells is a controversial issue. It has been reported that EGF stimulation disrupts cell–cell junctions and induces EMT in A549 cells [65, 66]. On the other hand, other reports have indicated no alteration in E-cadherin and vimentin levels upon EGF stimulation in A549 cells [67, 68]. Consistent with these reports, the decrease of E-cadherin and the increase of vimentin induced by EGFR ligand stimulation were less prominent than that by IL-1β (Fig. 6C–E). In addition, migratory potential was not sufficiently increased by only EGFR ligand stimulation compared with IL-1β (Fig. 6F). On the basis of these results, we speculated that in addition to the EGFR-dependent PI3K/AKT pathway, an EGFR-independent signaling pathway is required for the induction of IL-1β-mediated partial EMT, and found that the activation of the IL-1R-dependnet MEK/ERK pathway is required for the induction of the partial EMT-like phenotype. Interestingly, previous studies show that the intranasal instillation of IL-1β induces pulmonary fibrosis and the EMT of alveolar epithelial cells, which might be associated with the activation of PI3K/AKT pathway [69, 70]. In addition, a study confirms that inhibition of ERK1/2 attenuates bleomycin-induced pulmonary fibrosis, in which IL-1β plays a key role, by inhibiting the EMT induction [71]. Considering our observations and those of others, PI3K/AKT and MEK/ERK pathways might be a valid target for therapeutic intervention in lung fibrosis associated with IL-1β-mediated EMT.

The MEK/ERK pathway plays a central role in the regulation of various physiological processes such as cell proliferation, survival, and motility [72]. In general, activation of the MEK/ERK pathway is initiated by growth factor-dependent and Ras-mediated activation of Raf MAPK kinase kinases (MAP3Ks). Then, activated Raf phosphorylates and activates MEK1/2, which in turn activates ERK1/2 phosphorylation [73]. On the other hand, this Ras/Raf/MEK/ERK pathway is also considered a signaling pathway for promoting EMT; it represses the expression of epithelial proteins and increases the expression of mesenchymal proteins, thereby facilitating cell motility and invasion [74]. Apart from the activation by Raf, the MEK/ERK pathway can also be activated by other MAP3Ks, such as Tpl2 (also known as MAP3K8 and COT), Mos, and MAST1 [75]. Among them, Tpl2 is the only MAP3K that activates ERK1/2 in response to IL-1 stimulation [76], and its activity is controlled by IKKβ [44, 45]. The Ras/Raf/MEK/ERK pathway plays vital roles in cell proliferation, survival, and differentiation, whereas the IKKβ/Tpl2/MEK/ERK pathway regulates the expression of pro-inflammatory mediators in response to various pro-inflammatory stimuli [77]. Our study suggests that the MEK/ERK pathway, required for the induction of IL-1β-mediated partial EMT, is activated not by the Ras/Raf cascade but by the IKKβ/Tpl2 cascade because ERK phosphorylation was suppressed by the treatment with IKKβ and Tpl2 inhibitors (Fig. S7A) but not with EGFR TKIs (Fig. 7B). Although knowledge of Tpl2-involved EMT induction is limited, some reports have suggested a link between Tpl2 activation and organ fibrosis or cancer progression. For example, studies on Tpl2-deficient Tpl2^−/−^ mice have indicated that Tpl2 promotes liver fibrosis [78]. Furthermore, it has been reported that Tpl2 plays a role in cell transformation and metastasis in several cancers [79, 80]. Furthermore, IKKβ is essential for EMT induction and metastasis in breast cancer cells [81]. Considering our observations and those of others, the activation of the MEK/ERK pathway induced by IL-1β is thought to be triggered through the IKKβ/Tpl2 cascade and be involved in EMT-related disorders such as organ fibrosis and cancer metastasis.

The PI3K/AKT and MEK/ERK pathways have attracted widespread attention as a potential target for cancer therapy [82]. As mentioned above, both pathways are important in regulating cell proliferation, survival, and apoptosis. On the other hand, these are the two most frequently hyperactivated pathways in a wide range of human cancers, thereby suggesting their key role in carcinogenesis and cancer progression [83]. More importantly, broad crosstalk exists between the PI3K/AKT and MEK/ERK pathways [84]. In this study, we revealed that IL-1β stimulation activated both the PI3K/AKT and the MEK/ERK pathways via EGFR and IL-1R, respectively, and induced the partial EMT-like phenotype (Fig. 9). In contrast to previous reports, however, obvious crosstalk between the PI3K/AKT and MEK/ERK pathways was not confirmed (Fig. 8F), suggesting that the activation of both pathways occurs not sequentially but rather simultaneously for the induction of IL-1β-mediated partial EMT. On the other hand, our results agree with those of a previous study showing that IL-1β-mediated MMP-9 secretion required the activation of both PI3K/AKT and MEK/ERK pathways, and the activation of either of these pathways alone is not sufficient [85]. The reason behind this discrepancy likely lies the difference in the activation processes of the MEK/ERK pathway. Crosstalk between the PI3K/AKT and MEK/ERK pathways is known to be mediated by Ras and Raf, which can lead to cross-activation or cross-inhibition as well as pathway convergence [86]. For example, activated Ras recruits and activates Raf, inducing the MEK/ERK pathway. The activated Ras also recruits and activates the PI3K/AKT pathway by direct contact with the PI3K catalytic domain [87]. In contrast, AKT negatively regulates the ERK activation by phosphorylating inhibitory sites of the regulatory domain of Raf [88]. On the other hand, our results indicate that the IL-1β-mediated activation of the MEK/ERK pathway is controlled by the IKKβ/Tpl2 cascade and probably independent of the Ras/Raf cascade. This is supported by a recent study demonstrating that Tpl2 activates the MEK/ERK pathway independently of Raf [89]. Unfortunately, this may further complicate therapeutic strategies for the prevention and treatment of EMT-related disorders. Besides the IL-1R stimulation, the PI3K/AKT pathway is activated downstream of RTK and GPCR activation, whereas the IKKβ/Tpl2/MEK/ERK pathways are also activated downstream of Toll-like receptors and TNF receptor stimulation [90, 91]. Thus, the inhibition of only one pathway is thought to be insufficient for the treatment of EMT-related disorders. Considering these observations, dual inhibitions of the PI3K/AKT and MEK/ERK pathways are thought to provide clinical benefits for the prevention and treatment of EMT-related disorders such as organ fibrosis and cancer progression.

## Conclusions

In conclusion, we found that the activation of the PI3K/AKT and MEK/ERK signaling pathways by IL-1β coordinately promotes partial EMT induction. The former is downstream of the EGFR signaling pathway, whereas the latter is downstream of the IL-1R signaling pathway. Therapeutic agents that block these two pathways operating as a complex network are desired.

### Supplementary Information

Supplementary Material 1

## Data Availability

No datasets were generated or analysed during the current study.

## Abbreviations

ADAM: A disintegrin and metalloproteinase
AKT: Protein kinase B
AREG: Amphiregulin
CREB: CAMP response element binding protein
EGF: Epidermal growth factor
EGFR: Epidermal growth factor receptor
EMT: Epithelial–mesenchymal transition
ERK: Extracellular signal-regulated protein kinase
GPCR: G-protein coupled receptor
IL-1β: Interleukin-1β
IL-1R: IL-1 receptor
IκBα: Inhibitor of kappa-B α
IKK: IκB kinase
JAK: Janus kinase
JNK: C-jun N-terminal kinase
MAPK: Mitogen-activated protein kinase
MEK: Mitogen-activated and extracellular signal-regulated kinase kinase
MMP: Matrix metalloproteinase
MSK: Mitogen and stress-activated kinase
NF-κB: Nuclear factor kappa-B
NSCLC: Non-small cell lung cancer
PI3K: Phosphoinositide 3-kinase
RSK: P90 ribosomal S6 kinase
RTK: Receptor tyrosine kinase
STAT: Signal transducer and activator of transcription
TGF-β: Transforming growth factor-β
TKI: Tyrosine kinase inhibitor
TNF-α: Tumor necrosis factor-α
Tpl2: Tumor progression locus 2
