# Supplementary material for: IL-1β-activated PI3K/AKT and MEK/ERK pathways coordinately promote induction of partial epithelial–mesenchymal transition
Source: Cell Commun Signal. 2024 Aug 8;22:392. doi: 10.1186/s12964-024-01775-8 (PMC11308217; doi:10.1186/s12964-024-01775-8)
Supplement: Supplementary file 1 — Supplementary Material 1 [file 12964_2024_1775_MOESM1_ESM.pdf]

Supplementary information

IL-1β-activated PI3K/AKT and MEK/ERK pathways coordinately promote induction of partial epithelial–mesenchymal transition

Yosuke Tabei[1,*] and Yoshihiro Nakajima[1]

[1]Health and Medical Research Institute, National Institute of Advanced Industrial Science and Technology (AIST), 2217-14 Hayashi-cho, Takamatsu, Kagawa 761-0395, Japan

[*]Corresponding author
Address: Health and Medical Research Institute, National Institute of Advanced Industrial Science and Technology (AIST), 2217-14 Hayashi-cho, Takamatsu, Kagawa 761-0395, Japan
E-mail: y-tabei@aist.go.jp
Tel: +81-80-2230-4245

Supplementary Figure S1

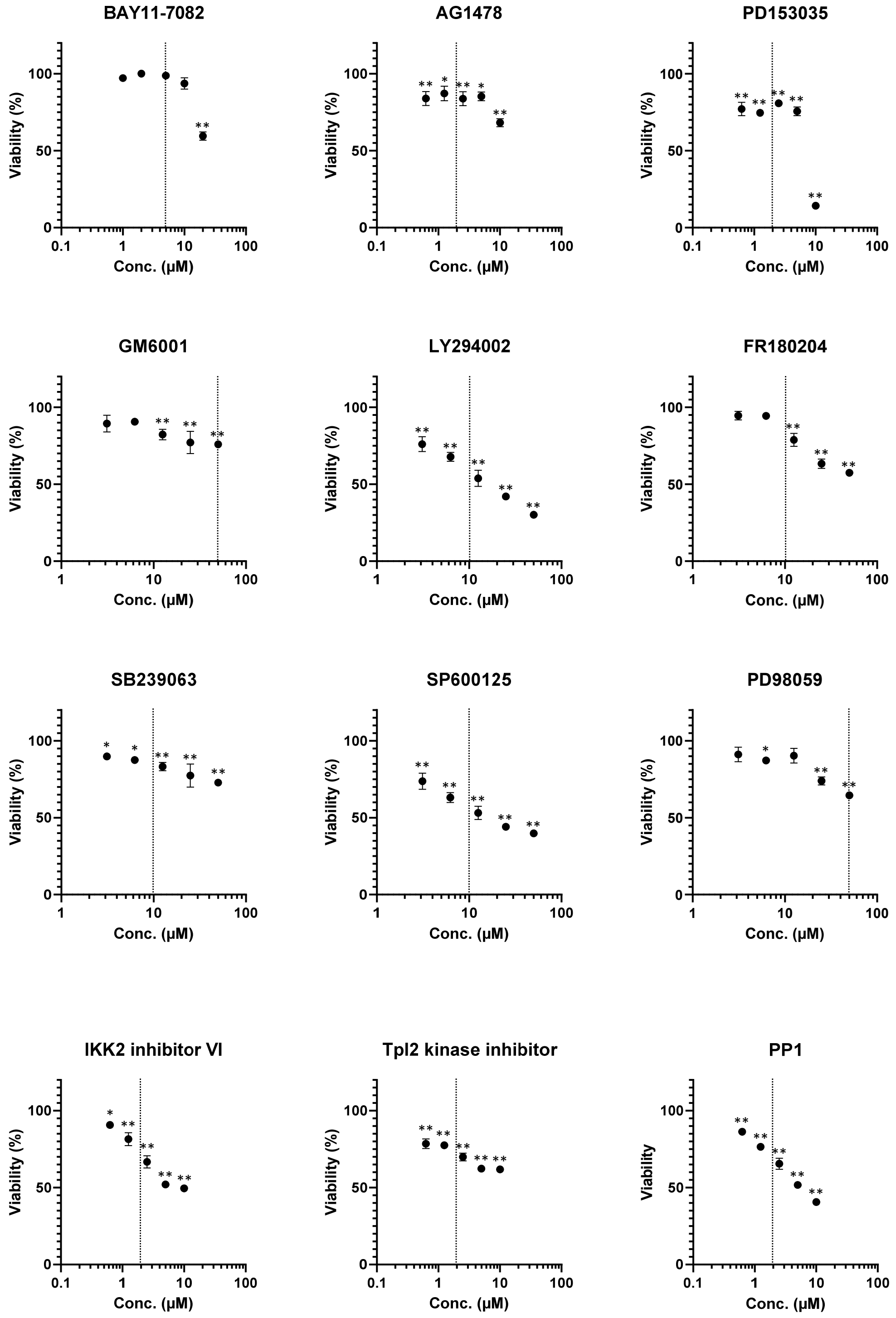

Supplementary Figure S1. Effects of chemical inhibitors on cell viability. A549 cells were treated with various concentrations of chemical inhibitors for 48 h. Cell viability was measured using the WST-1 assay, and the results are expressed as percentage of untreated controls. Values are means ± SD, n = 3, one-way ANOVA followed by Tukey's multiple comparison test. $*P < 0.05$, $**P < 0.01$, compared with untreated control. Vertical dashed lines indicate the maximum concentrations used in this study.

Supplementary Figure S2

Whole images of western blotting of Figure 1C.

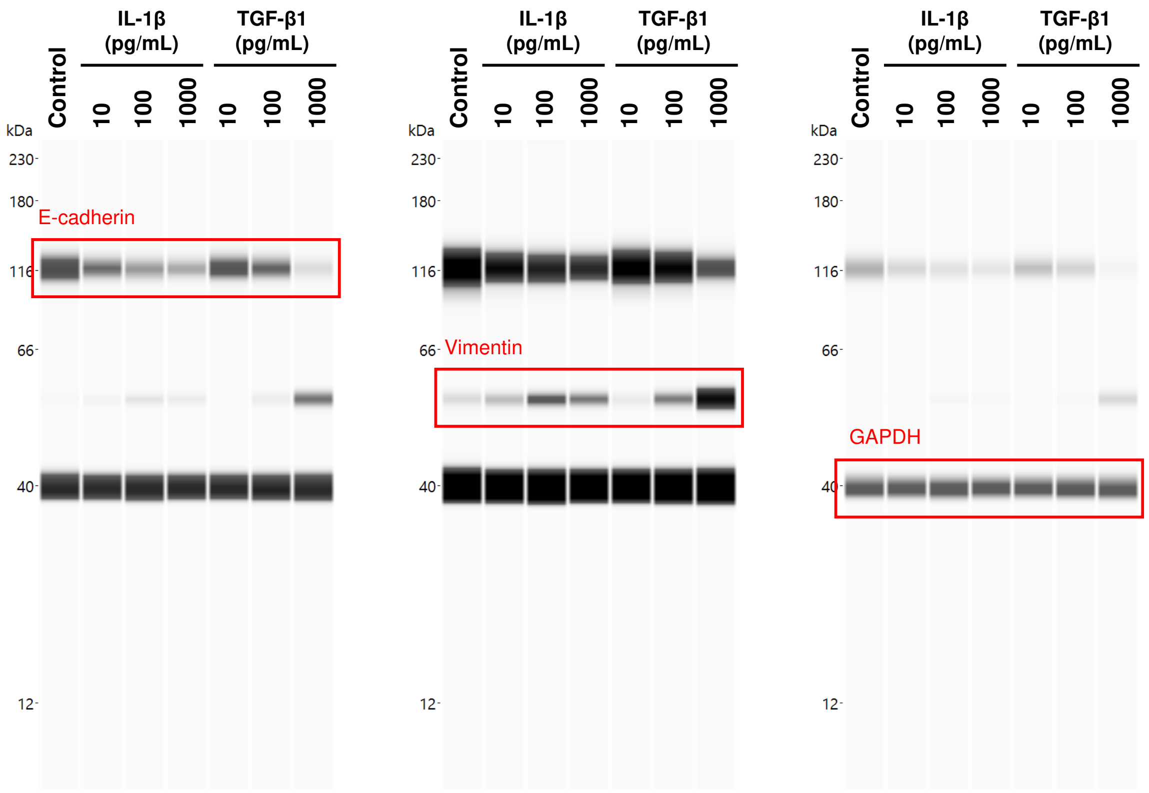

Whole images of western blotting of Figure 2A.

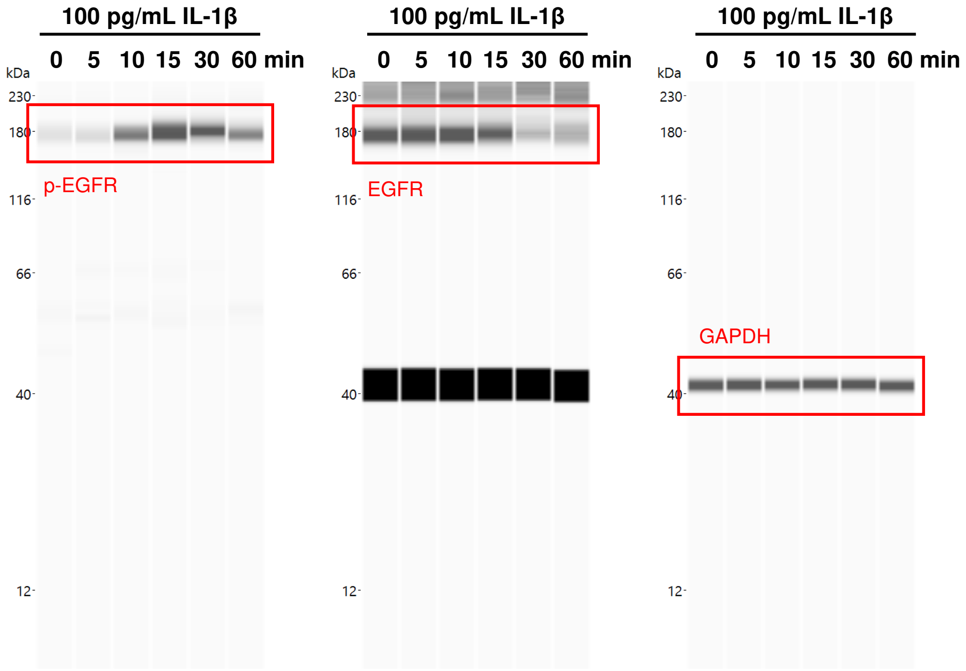

Supplementary Figure S2

Whole images of western blotting of Figure 2B.

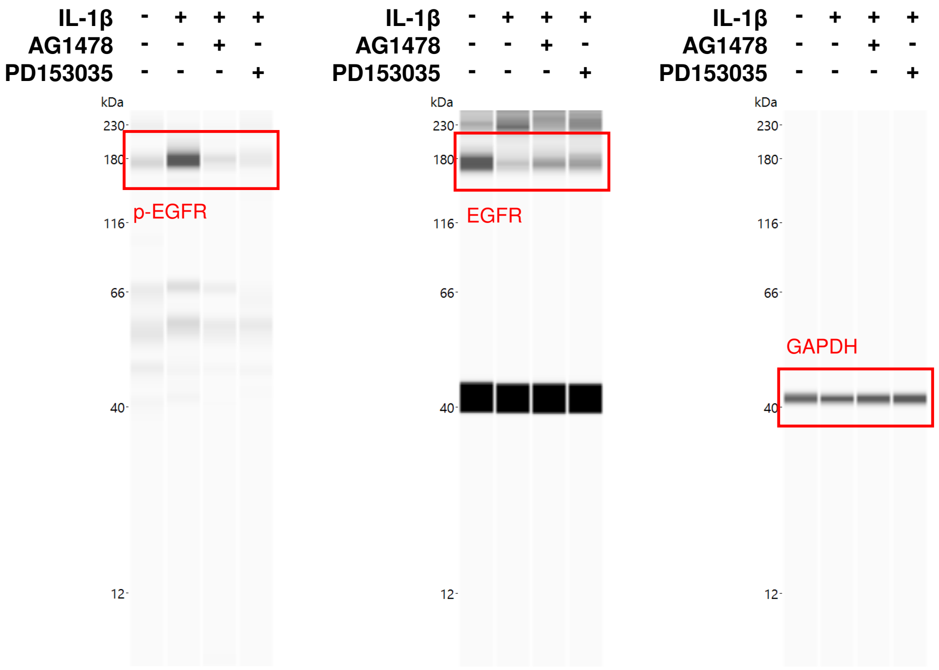

Whole images of western blotting of Figure 3B.

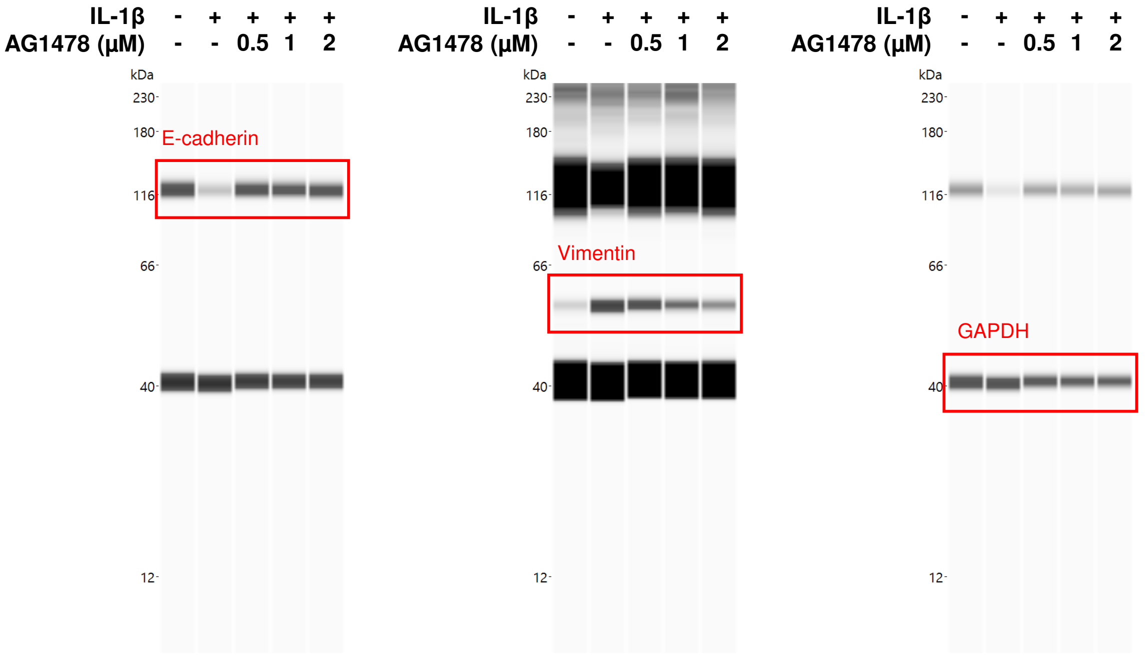

Supplementary Figure S2

Whole images of western blotting of Figure 3B.

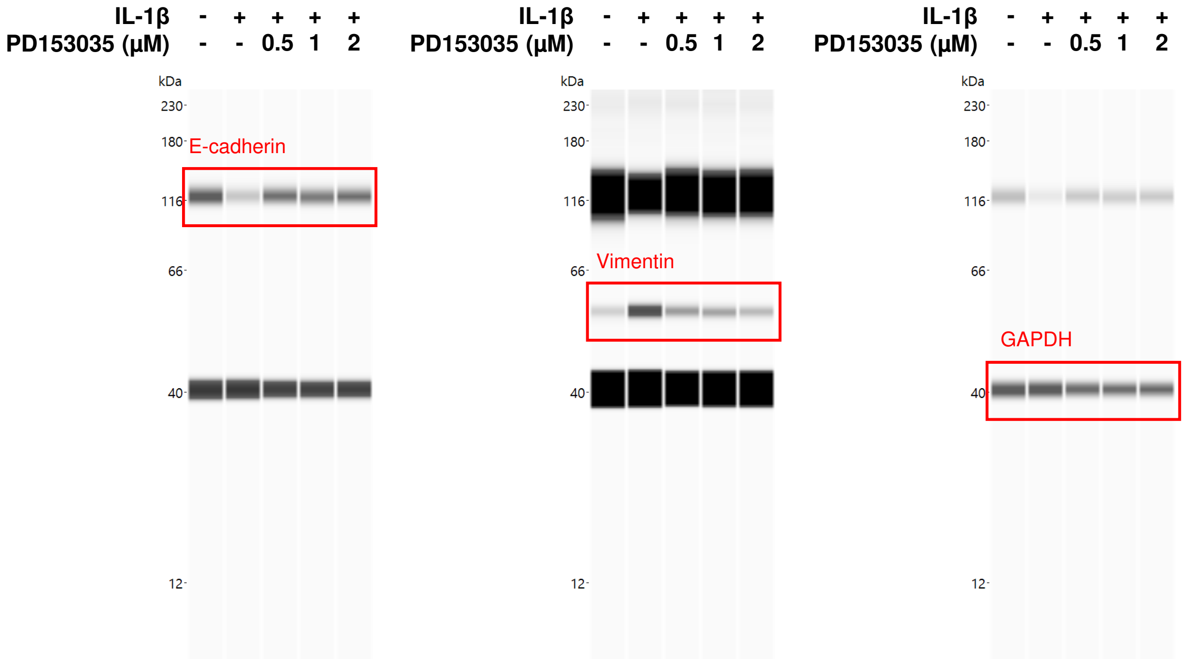

Whole images of western blotting of Figure 4B.

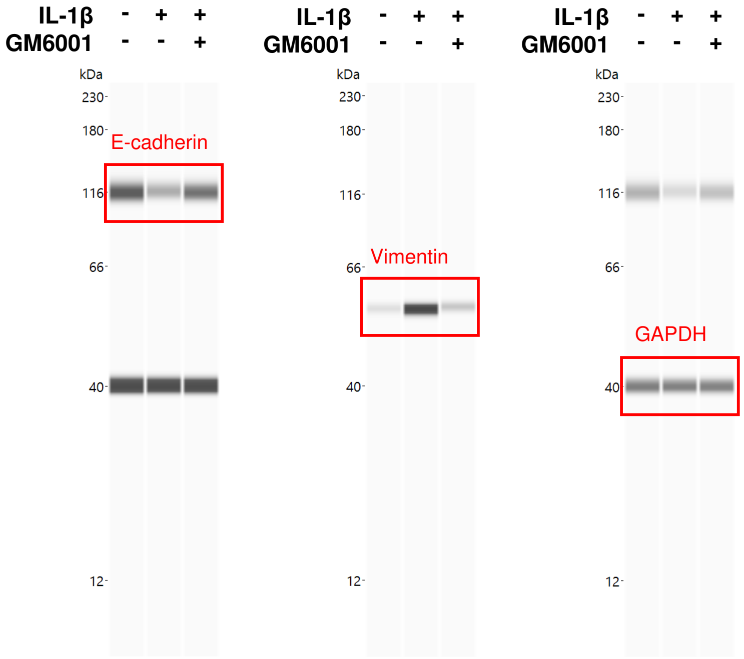

Supplementary Figure S2

Whole images of western blotting of Figure 4D.

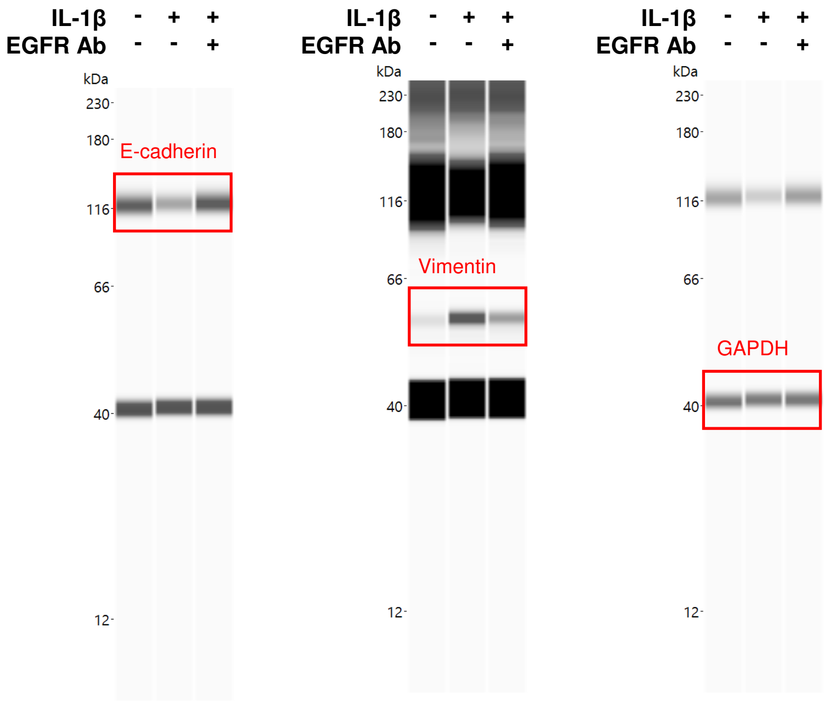

Whole images of western blotting of Figure 4G.

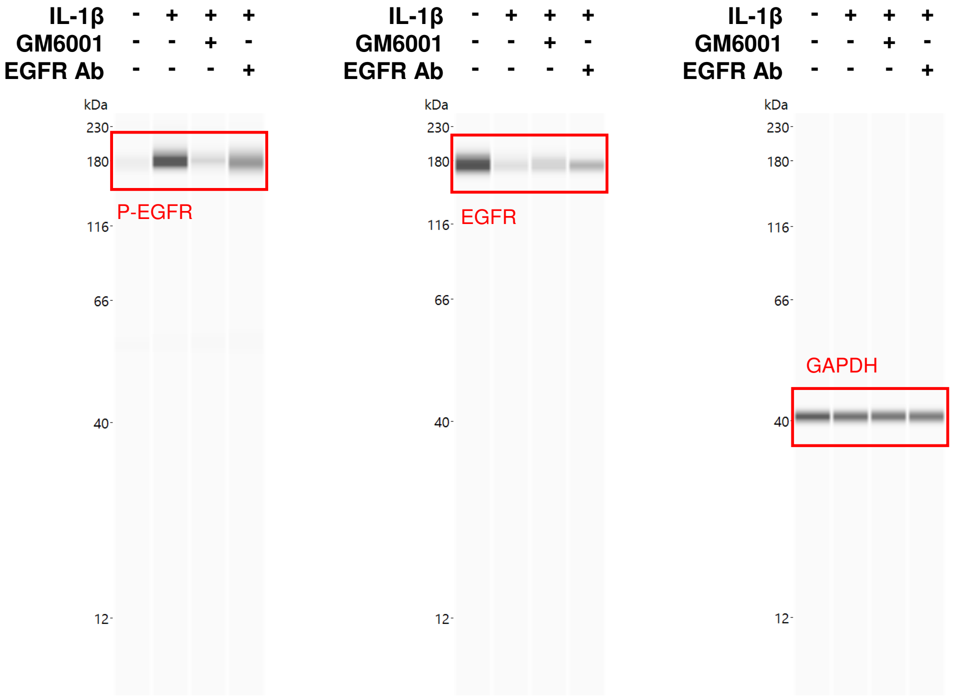

Supplementary Figure S2

Whole images of western blotting of Figure 5A.

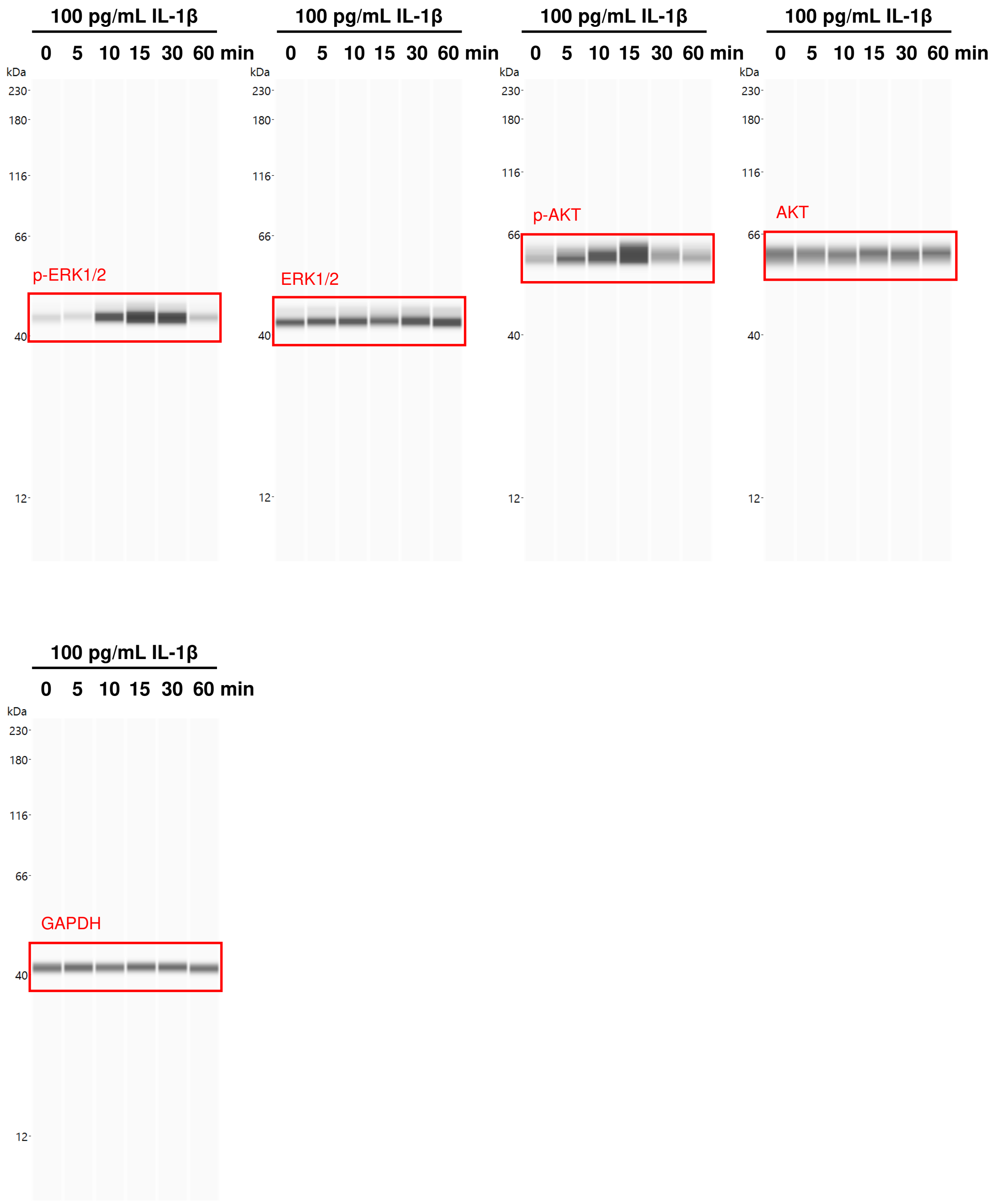

Supplementary Figure S2

Whole images of western blotting of Figure 5B.

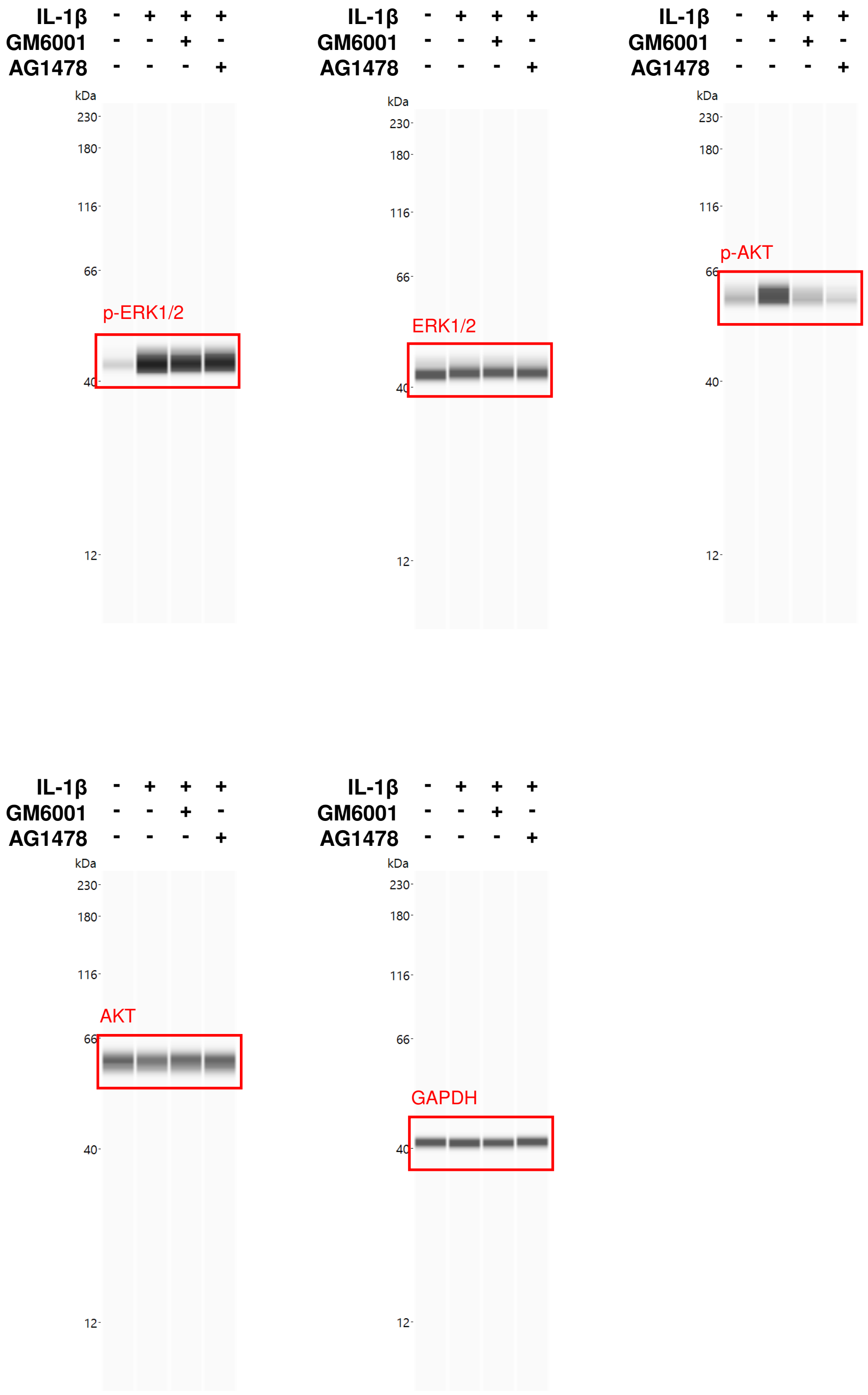

Supplementary Figure S2

Whole images of western blotting of Figure 5C.

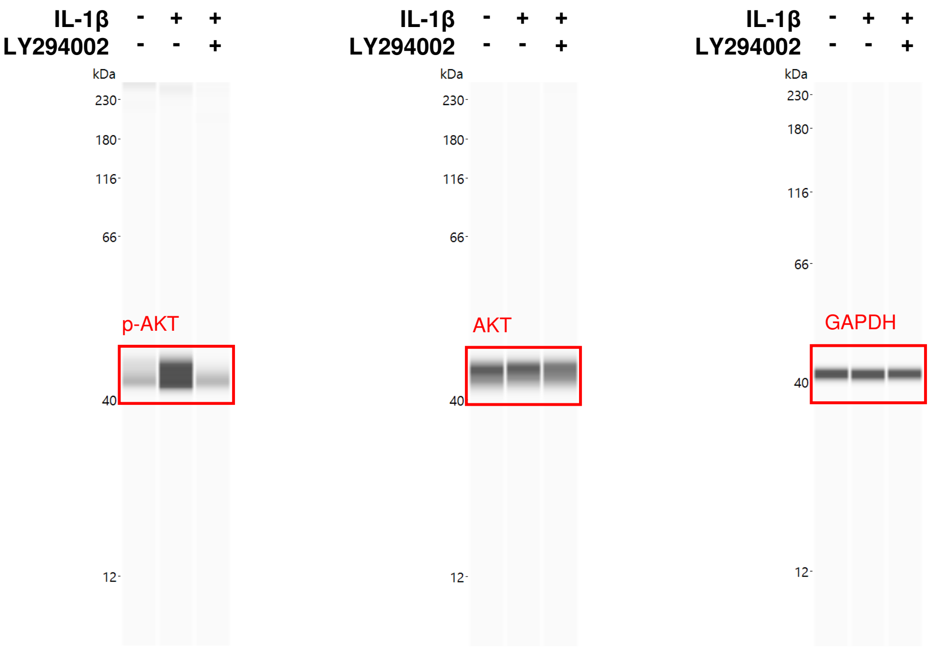

Whole images of western blotting of Figure 5E.

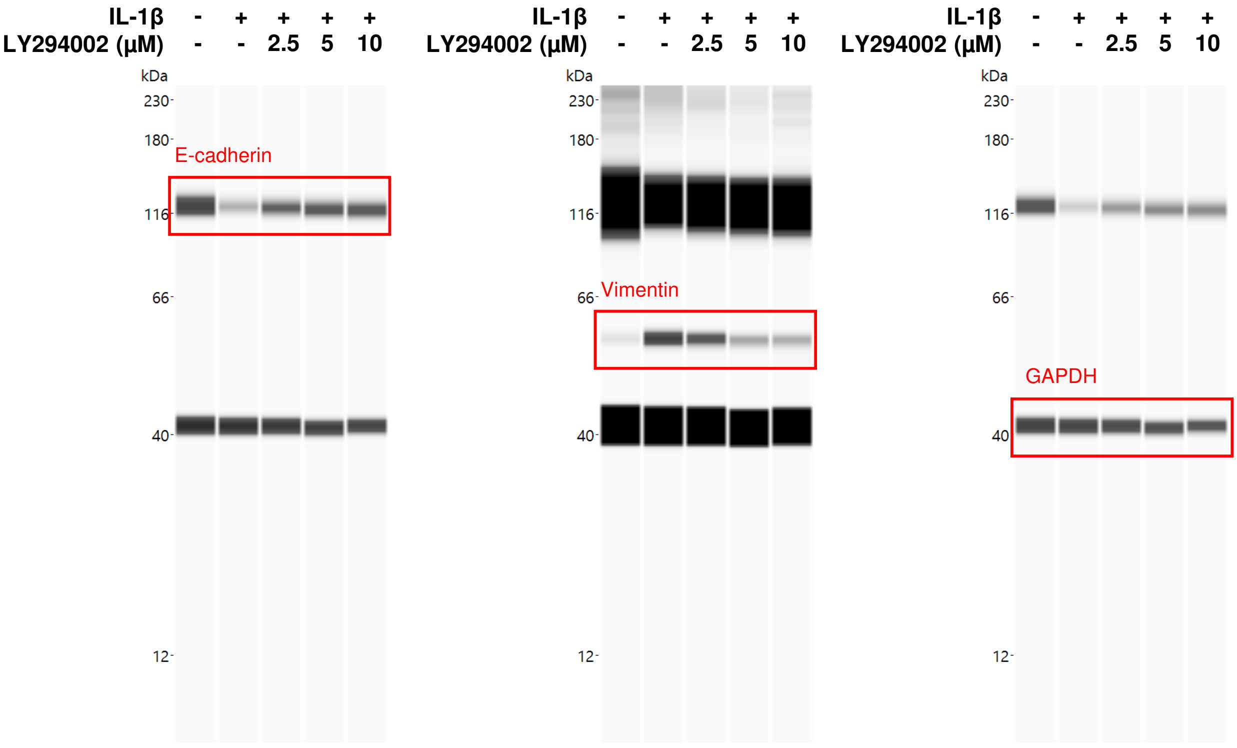

Supplementary Figure S2

Whole images of western blotting of Figure 6A.

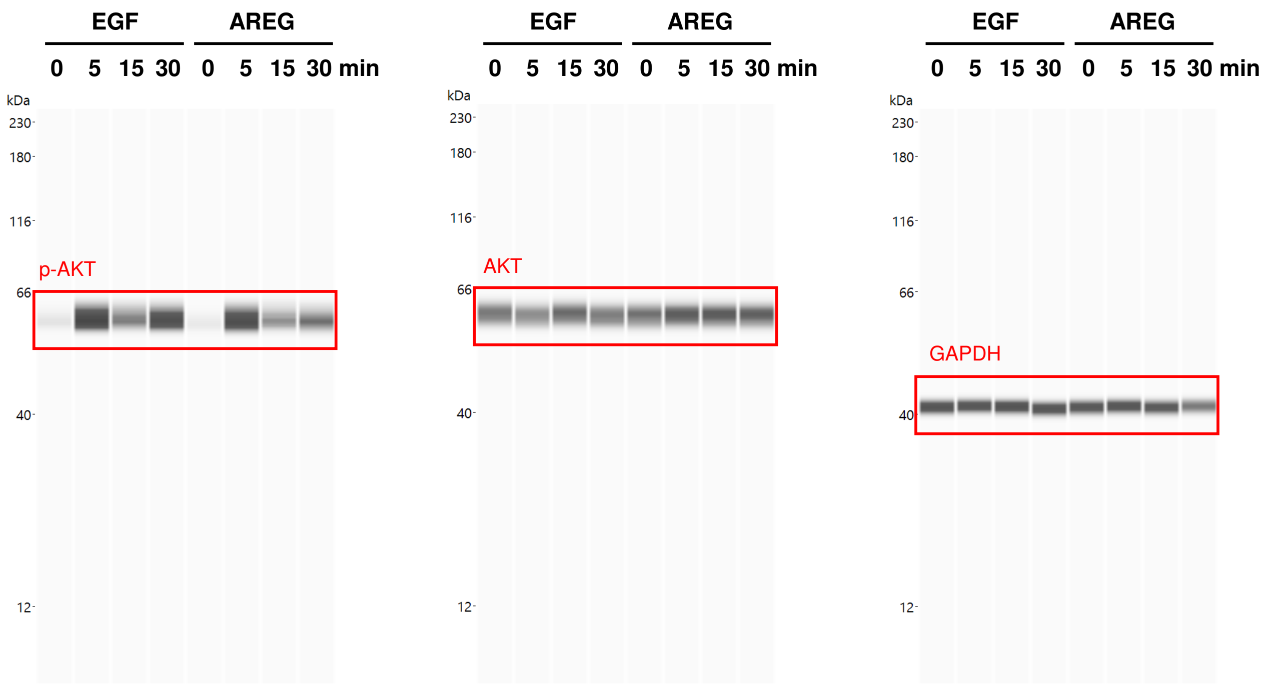

Whole images of western blotting of Figure 6B.

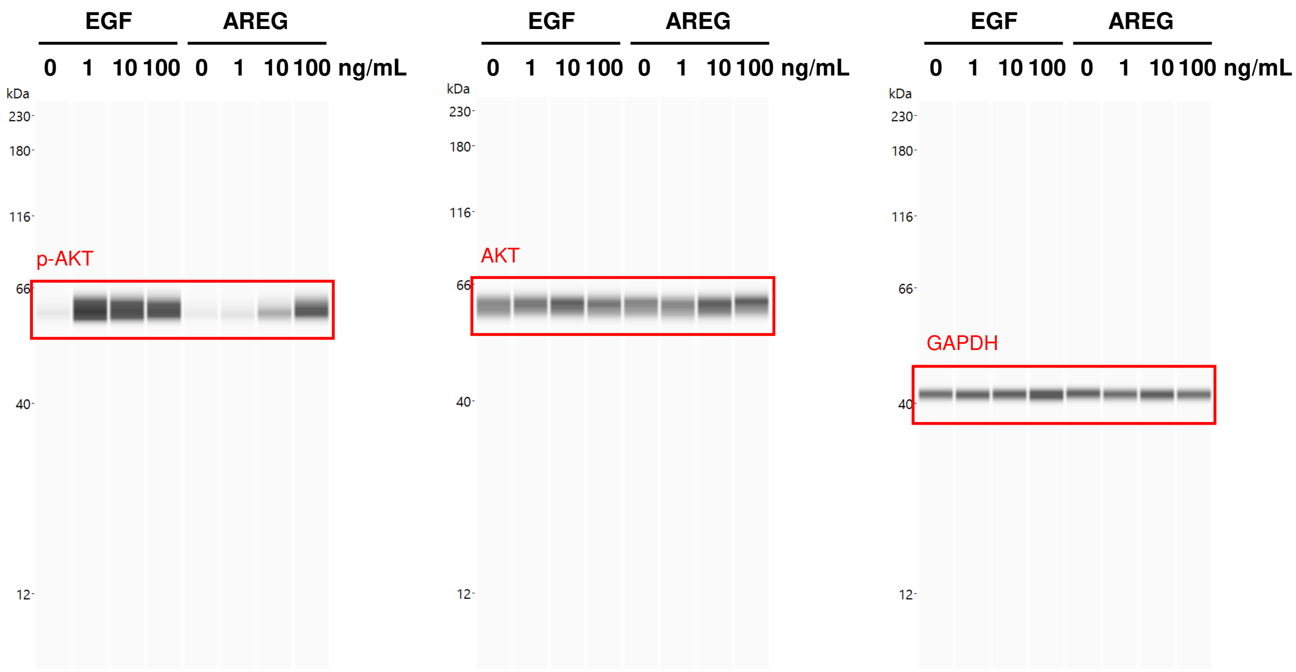

Supplementary Figure S2

Whole images of western blotting of Figure 6D.

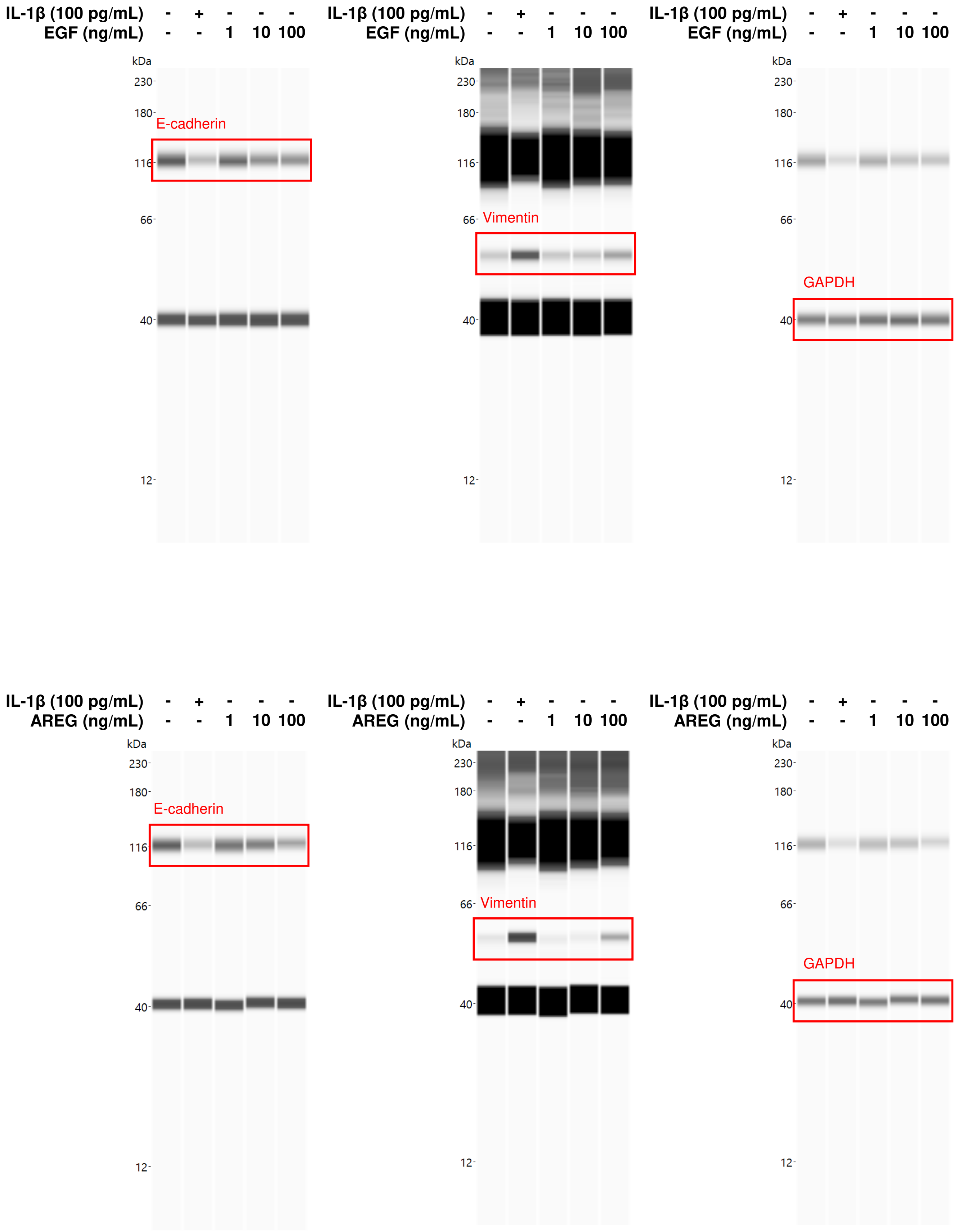

Supplementary Figure S2

Whole images of western blotting of Figure 7A.

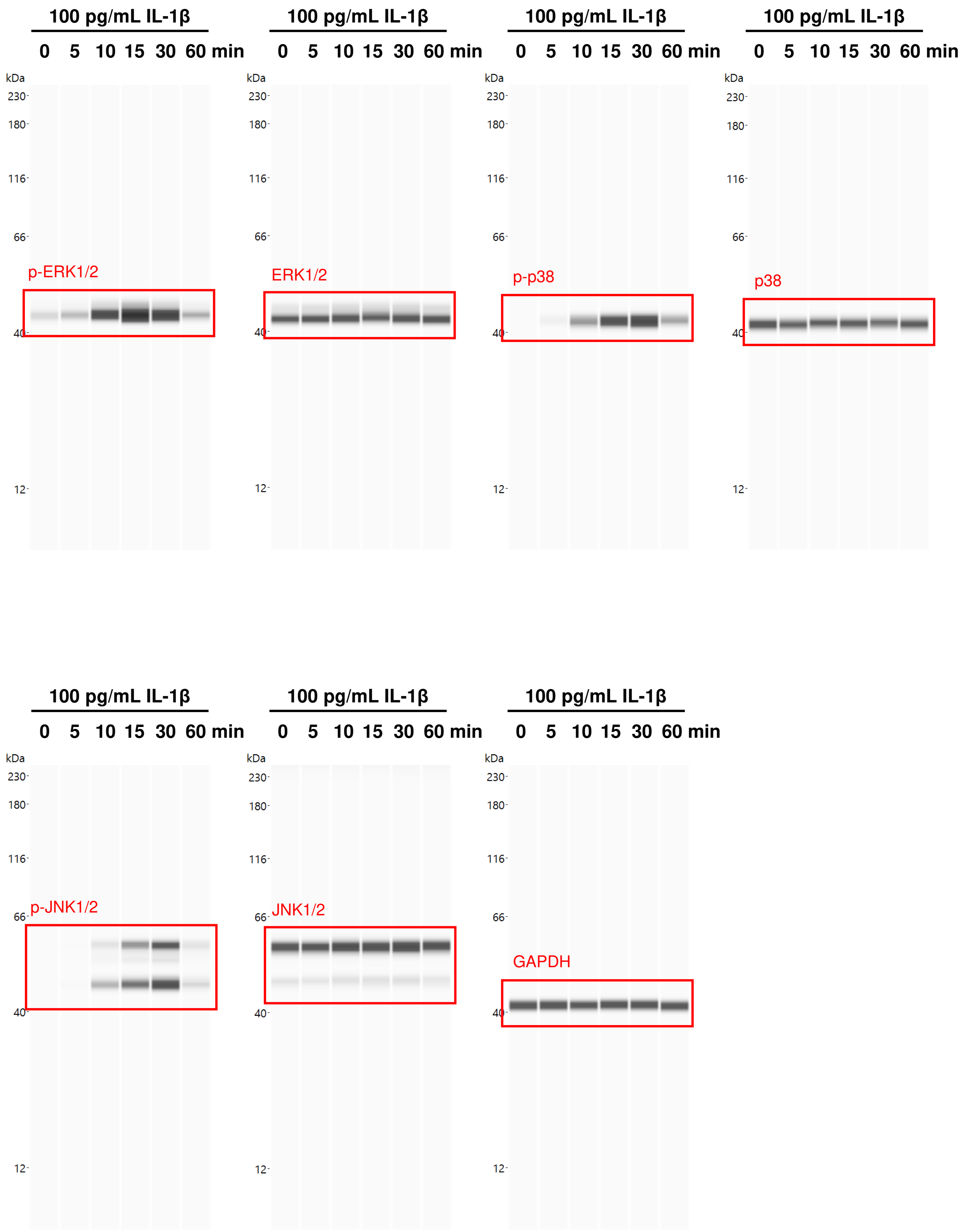

Supplementary Figure S2

Whole images of western blotting of Figure 7B.

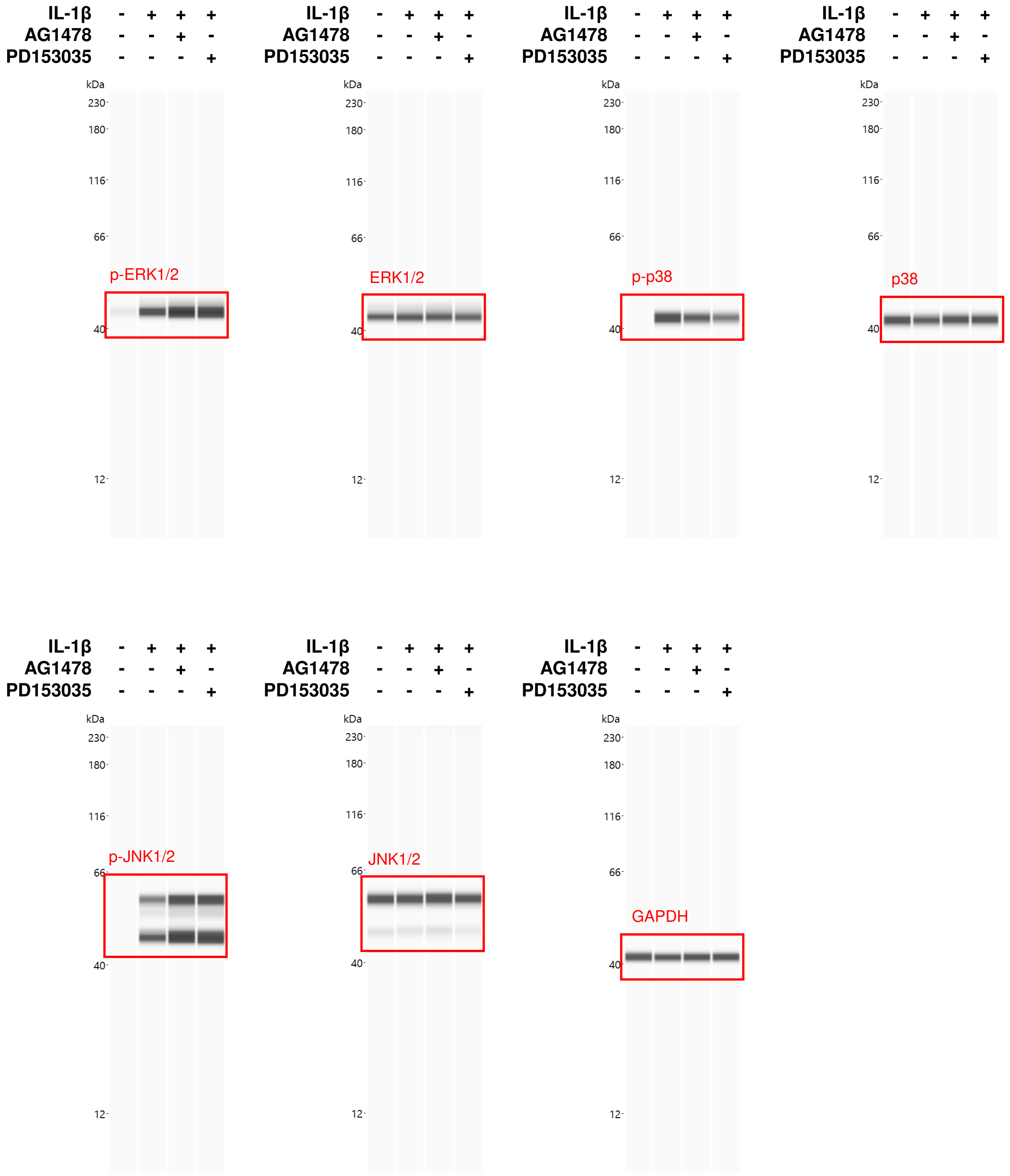

Supplementary Figure S2

Whole images of western blotting of Figure 7D.

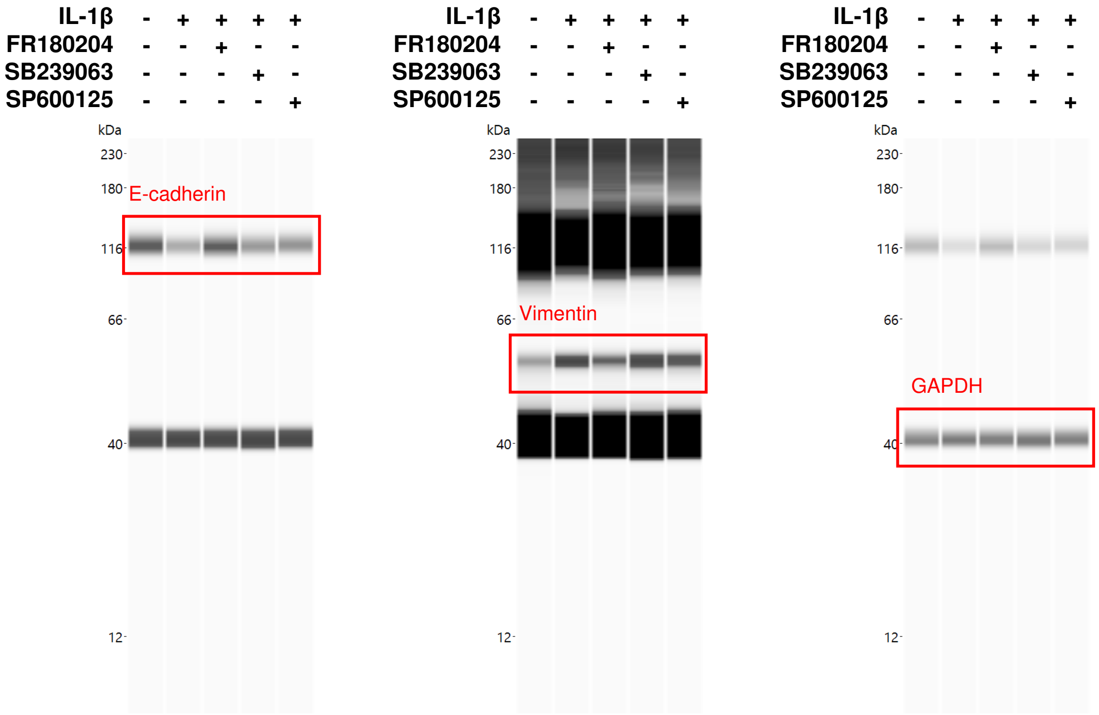

Whole images of western blotting of Figure 8B.

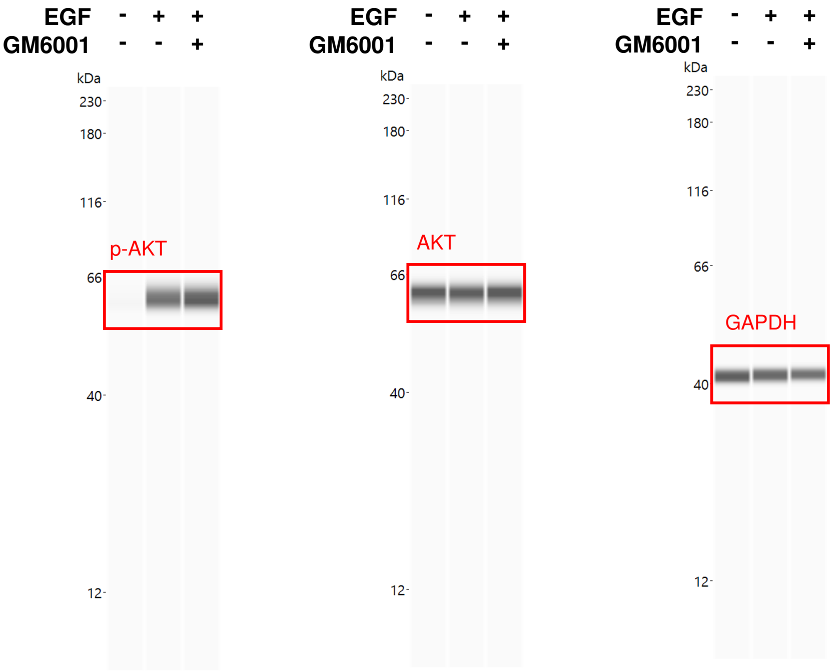

Supplementary Figure S2

Whole images of western blotting of Figure 8D.

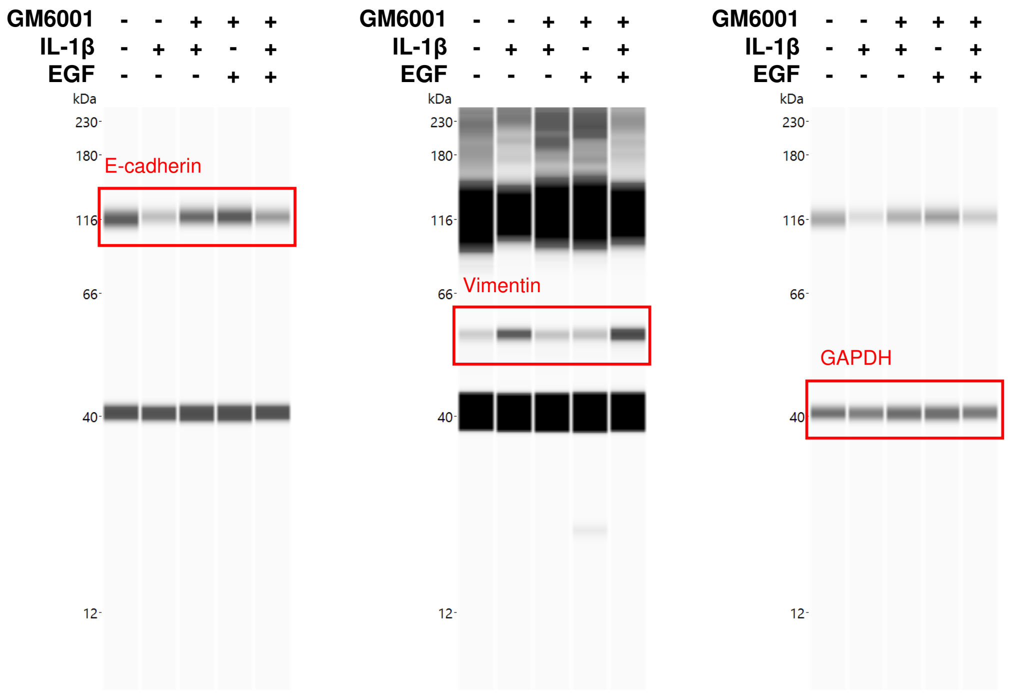

Whole images of western blotting of Figure 8G.

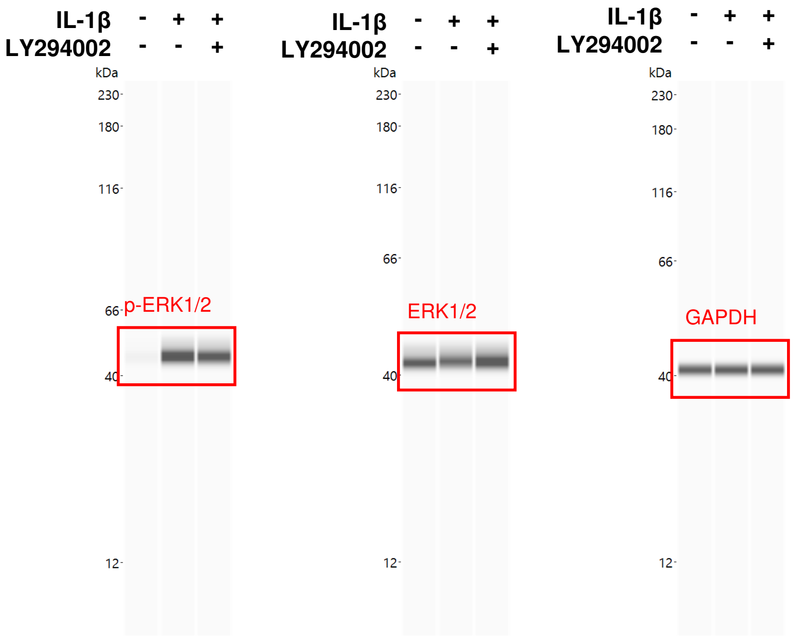

Supplementary Figure S2

Whole images of western blotting of Figure 8G.

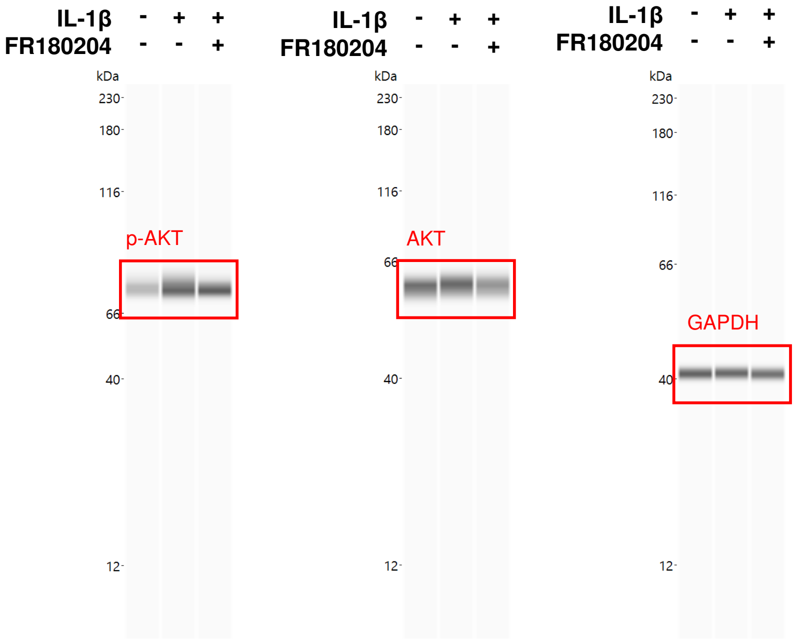

Supplementary Figure S2

Whole images of western blotting of Supplementary Figure S3.

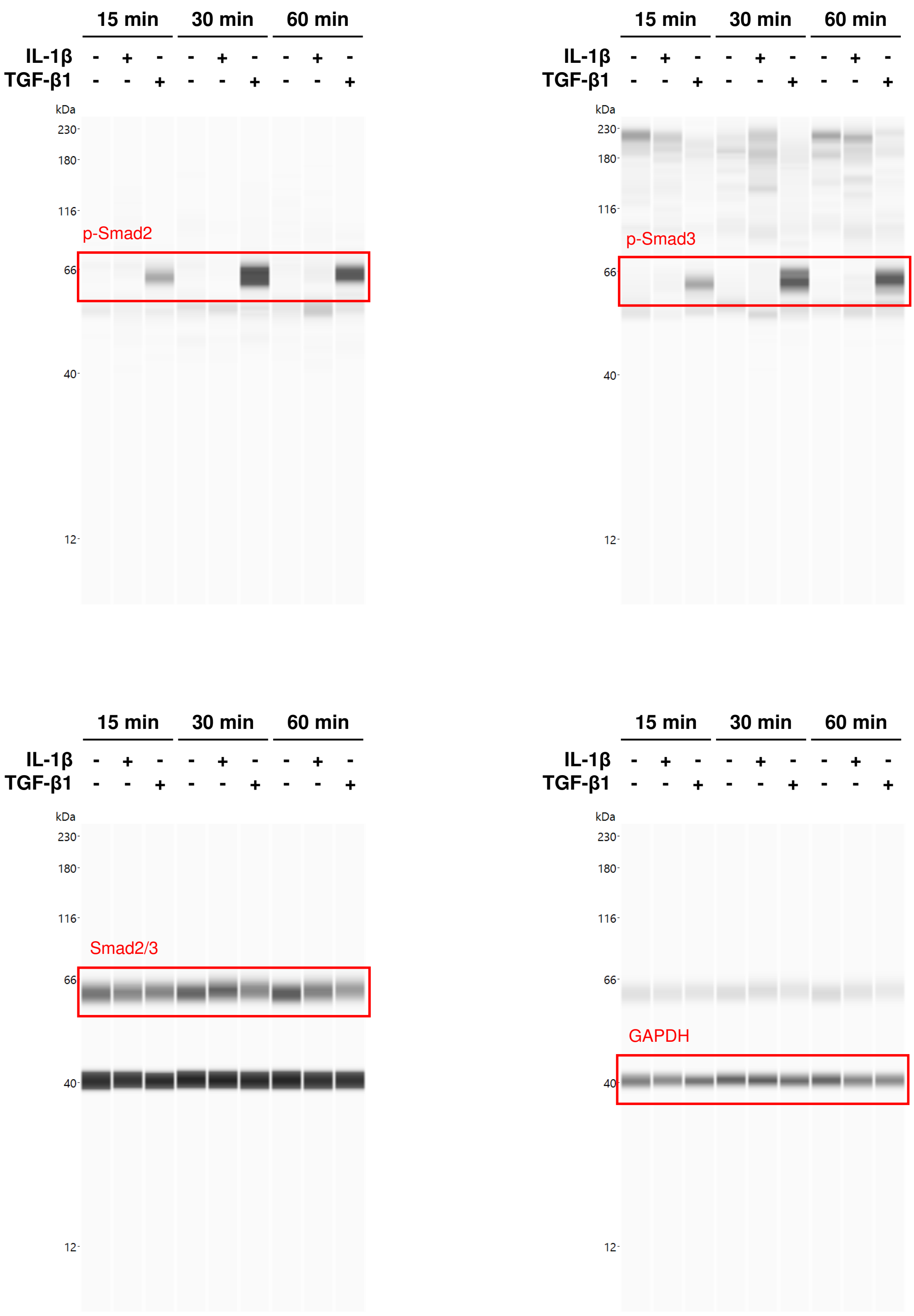

Supplementary Figure S2

Whole images of western blotting of Supplementary Figure S4A.

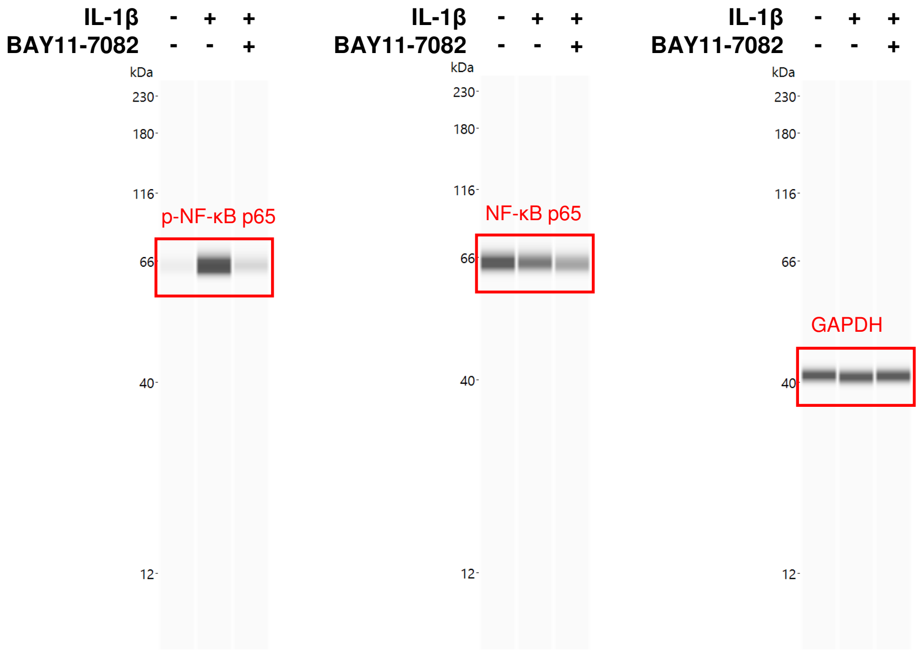

Whole images of western blotting of Supplementary Figure S4C.

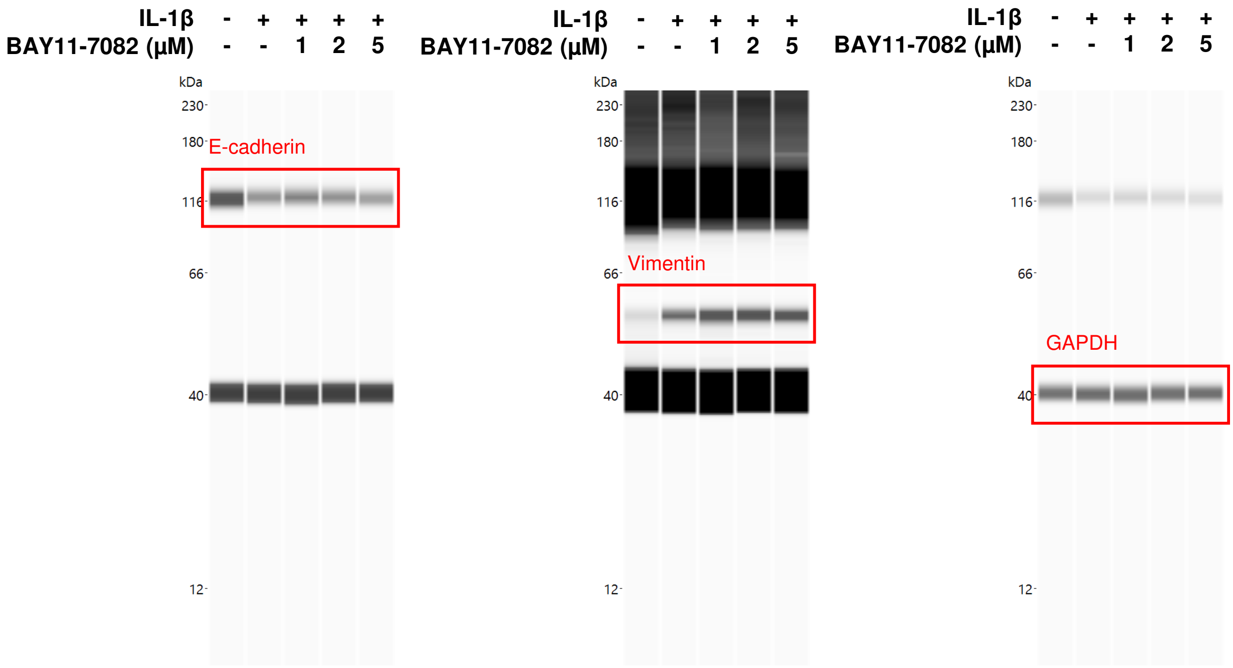

Supplementary Figure S2

Whole images of western blotting of Supplementary Figure S5A.

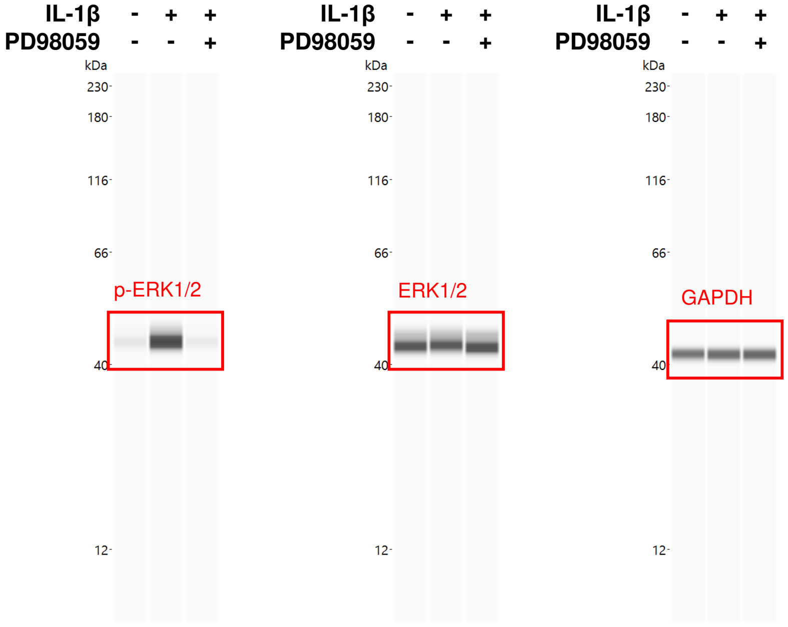

Whole images of western blotting of Supplementary Figure S5C.

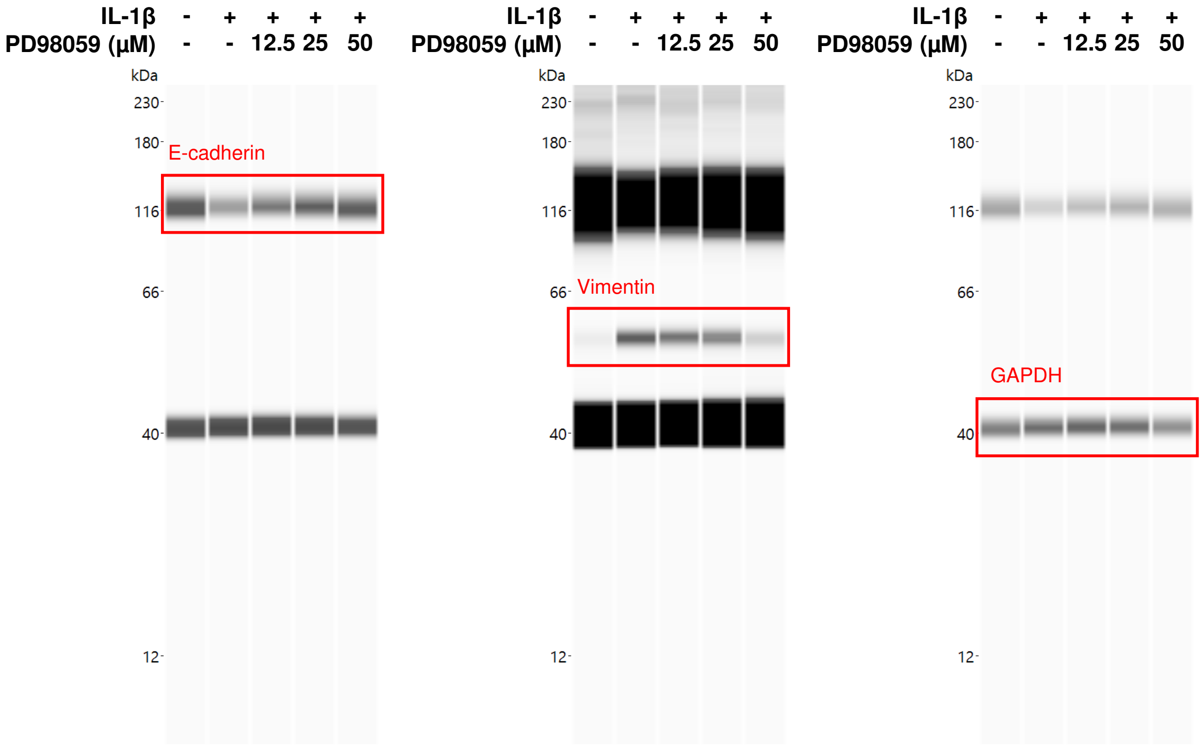

Supplementary Figure S2

Whole images of western blotting of Supplementary Figure S7A.

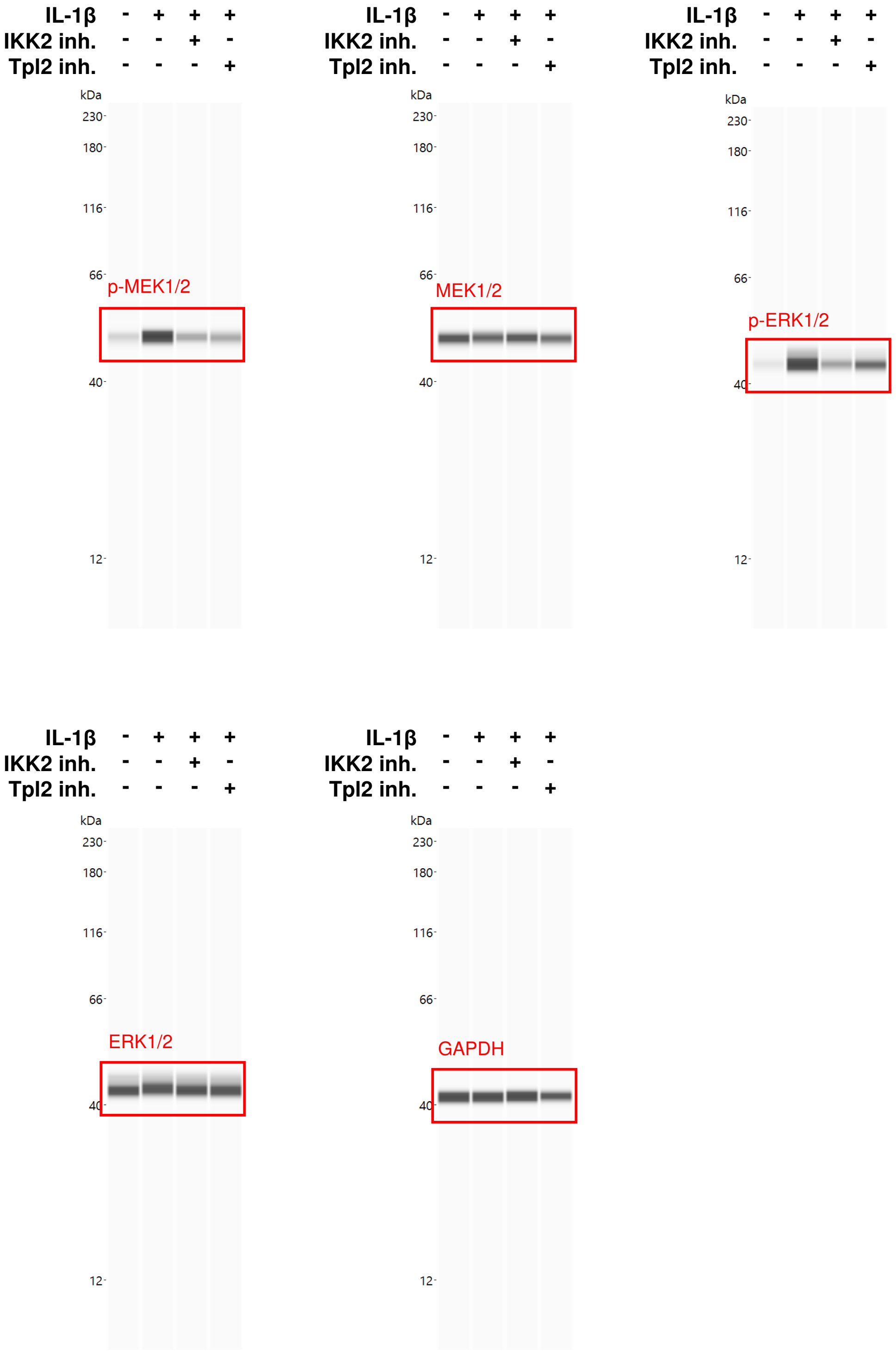

Supplementary Figure S2

Whole images of western blotting of Supplementary Figure S7C.

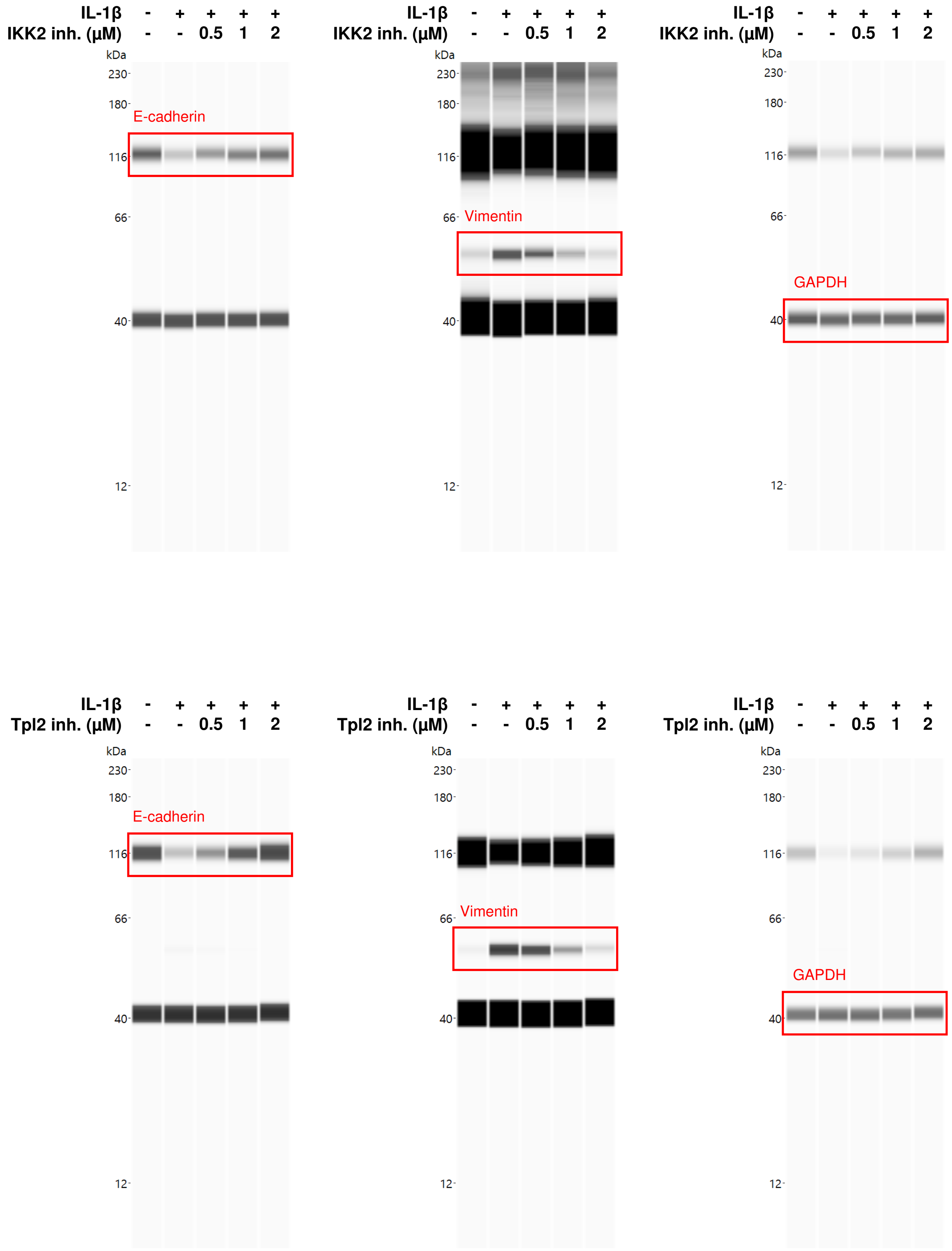

Supplementary Figure S2

Whole images of western blotting of Supplementary Figure S8.

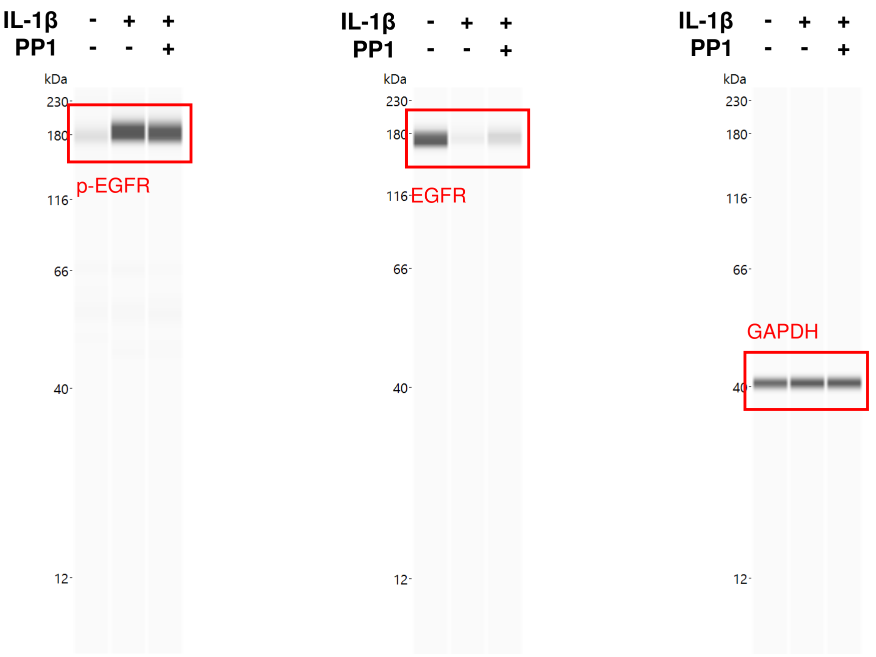

Supplementary Figure S2. Whole images of western blotting.

Supplementary Figure S3

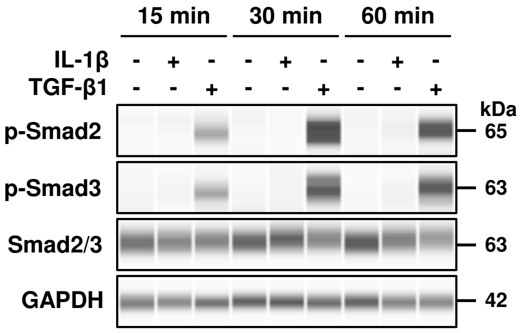

Supplementary Figure S3. Western blot analysis of Smad2 and 3 phosphorylation in A549 cells stimulated with 100 pg/mL IL-1β or 1000 pg/mL TGF-β1 for the indicated times. GAPDH was used as internal control.

Supplementary Figure S4

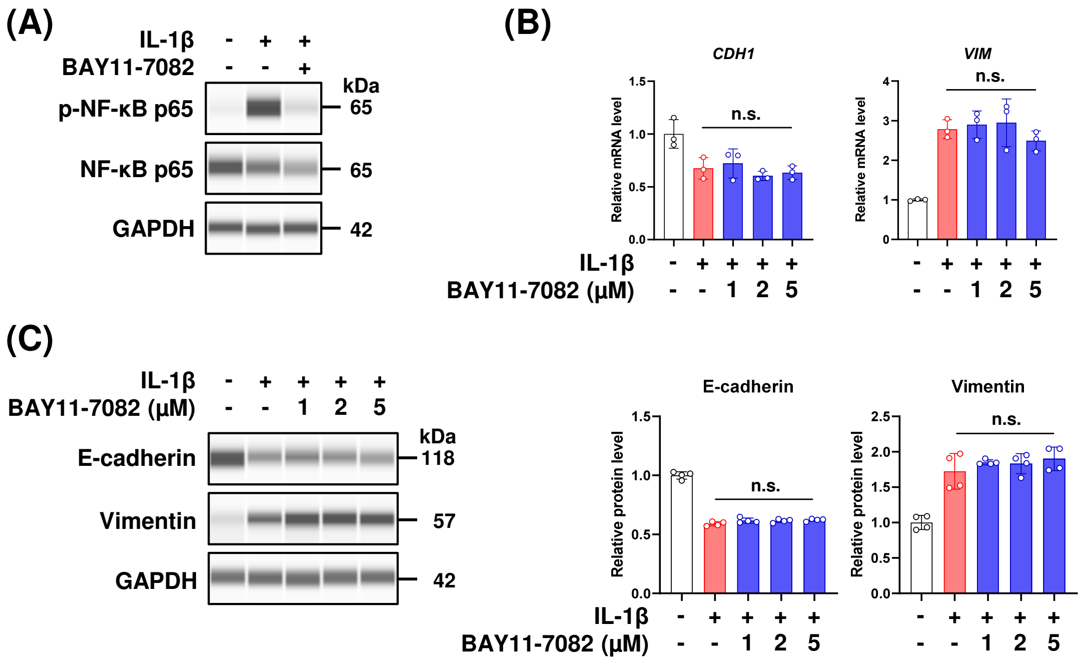

Supplementary Figure S4. Effect of IκBα kinase inhibitor BAY11-7082 on the induction of EMT in A549 cells stimulated with IL-1β. **A** Western blot analysis of NF-κB p65 phosphorylation. The cells were treated with 5 μM BAY11-7082 for 4 h and then stimulated with 100 pg/mL IL-1β for an additional 5 min. GAPDH was used as internal control. **B** *CDH1* and *VIM* mRNA expression levels. The cells were treated with 5 μM BAY11-7082 for 4 h and then stimulated with 100 pg/mL IL-1β for an additional 48 h. Each mRNA expression level was normalized to the corresponding *ACTB* value and is presented as relative units to untreated control. Values are means ± SD, n = 3, one-way ANOVA followed by Tukey's multiple comparison test. n.s., not significant, compared with IL-1β-treated cells. **C** Western blot analysis of E-cadherin and vimentin. The cells were treated with 5 μM BAY11-7082 for 4 h and then stimulated with 100 pg/mL IL-1β for an additional 48 h. Each protein level was normalized to the corresponding GAPDH value and is presented as relative units to untreated control. Values are means ± SD, n = 4, one-way ANOVA followed by Tukey's multiple comparison test. n.s., not significant, compared with IL-1β-treated cells.

Supplementary Figure S5

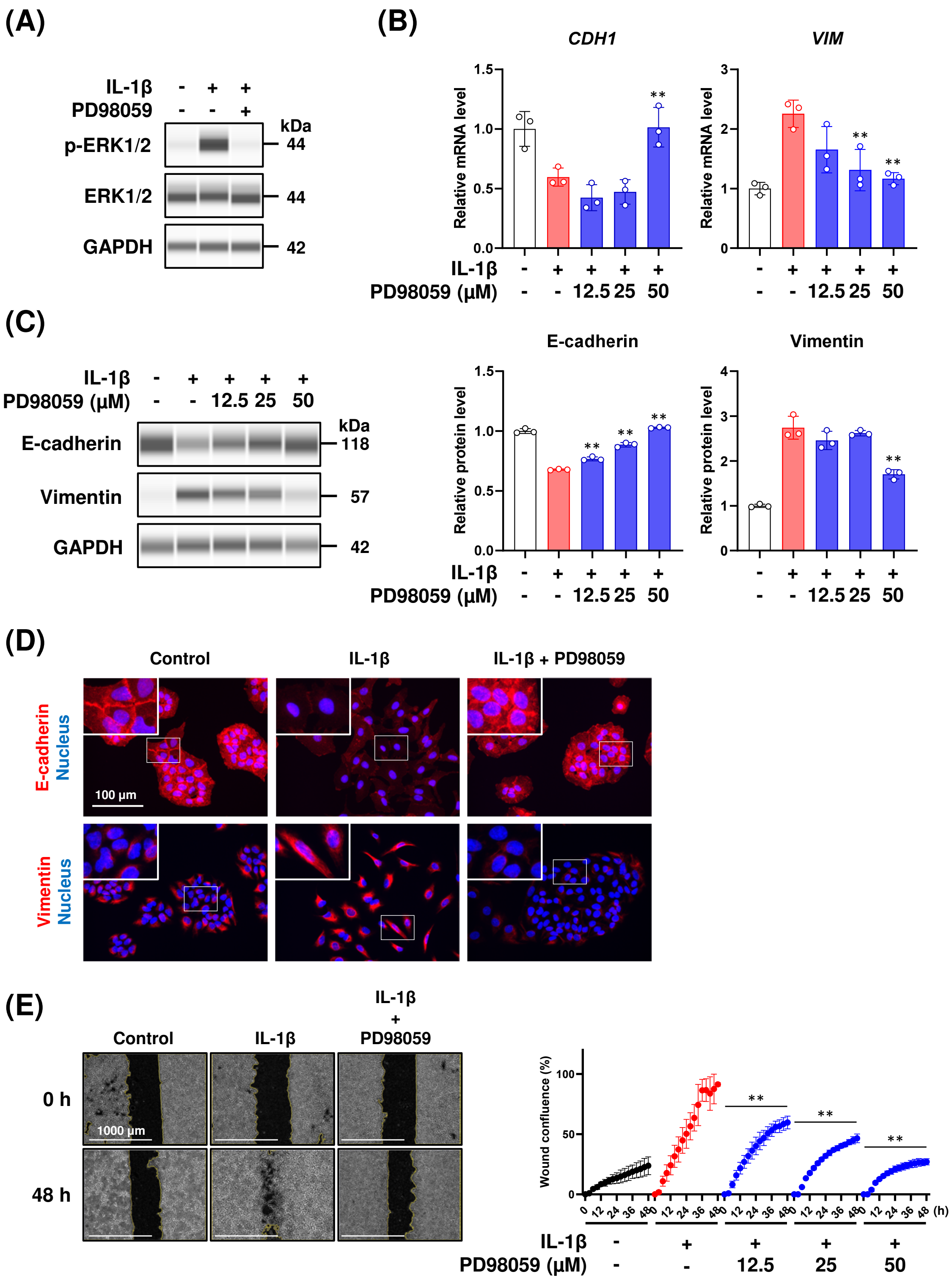

Supplementary Figure S5. Effect of MEK inhibitor PD98059 on the induction of EMT in A549 cells stimulated with IL-1β. **A** Western blot analysis of ERK1/2 phosphorylation in cells treated with IL-1β in the presence of PD98059 for 30 min. GAPDH was used as internal control. **B** *CDH1* and *VIM* mRNA expression levels in cells treated with IL-1β and PD98059 for 48 h. Each mRNA expression level was normalized to the corresponding *ACTB* value and is presented as relative units to untreated control. Values are means ± SD, n = 3, one-way ANOVA followed by Tukey's multiple comparison test. $^{**}P < 0.01$, compared with IL-1β-treated cells. **C** Western blot analysis of E-cadherin and vimentin in cells treated with IL-1β and PD98059 for 48 h. Each protein level was normalized to the corresponding GAPDH value and is presented as relative units to untreated control. Values are means ± SD, n = 4, one-way ANOVA followed by Tukey's multiple comparison test. $^{**}P < 0.01$, compared with IL-1β-treated cells. **D** Immunofluorescence analysis of E-cadherin (upper panels) and vimentin (bottom panels) in cells treated with IL-1β in the presence of PD98059 for 48 h. Nuclei were stained with NucBlue. Fluorescence images were obtained by using a BZ-X710 fluorescence microscope. Insets show high-magnification images of the boxed areas. **E** Wound healing assay of cells treated with IL-1β in the presence of PD98059. Images (upper panels) and wound confluence values (bottom panel) were obtained using a Lionheart FX automated live cell imager and Gen5 software, respectively. Values are means ± SD, n = 3, two-way ANOVA followed by Tukey's multiple comparison test. $^{**}P < 0.01$, compared with IL-1β-treated cells.

Supplementary Figure S6

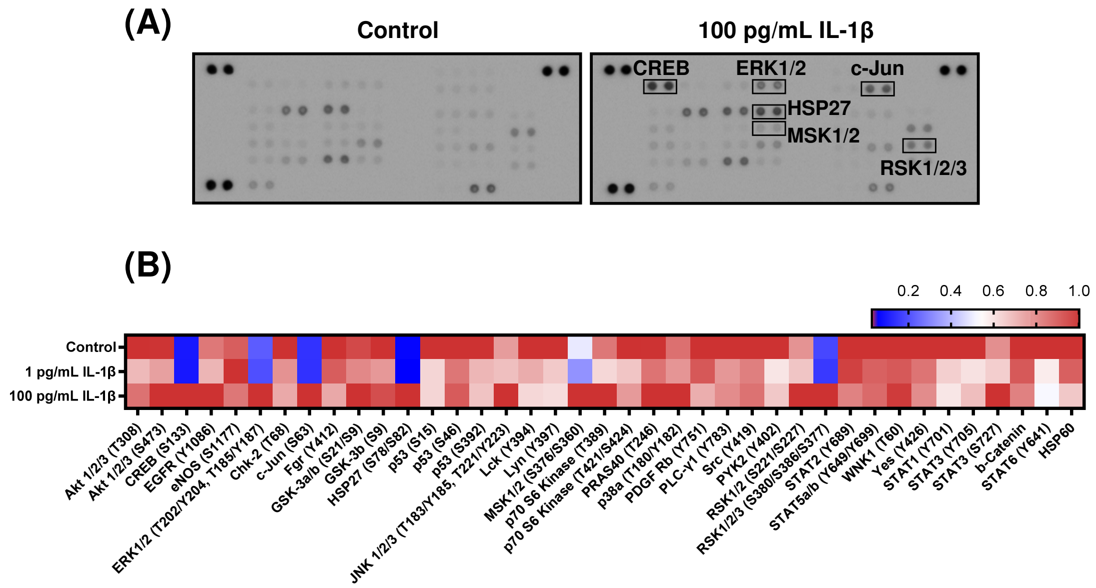

Supplementary Figure S6. Analysis of the kinase phosphorylation in A549 cells stimulated with IL-1β. **A** Representative image of phosphor-kinase arrays. **B** Heatmap of 39 phospho-kinases. The proteins were collected from the cells treated with IL-1β for 30 min and analyzed using a Proteome Profiler Human Phospho-Kinase Array Kit as described in Materials and Methods.

Supplementary Figure S7

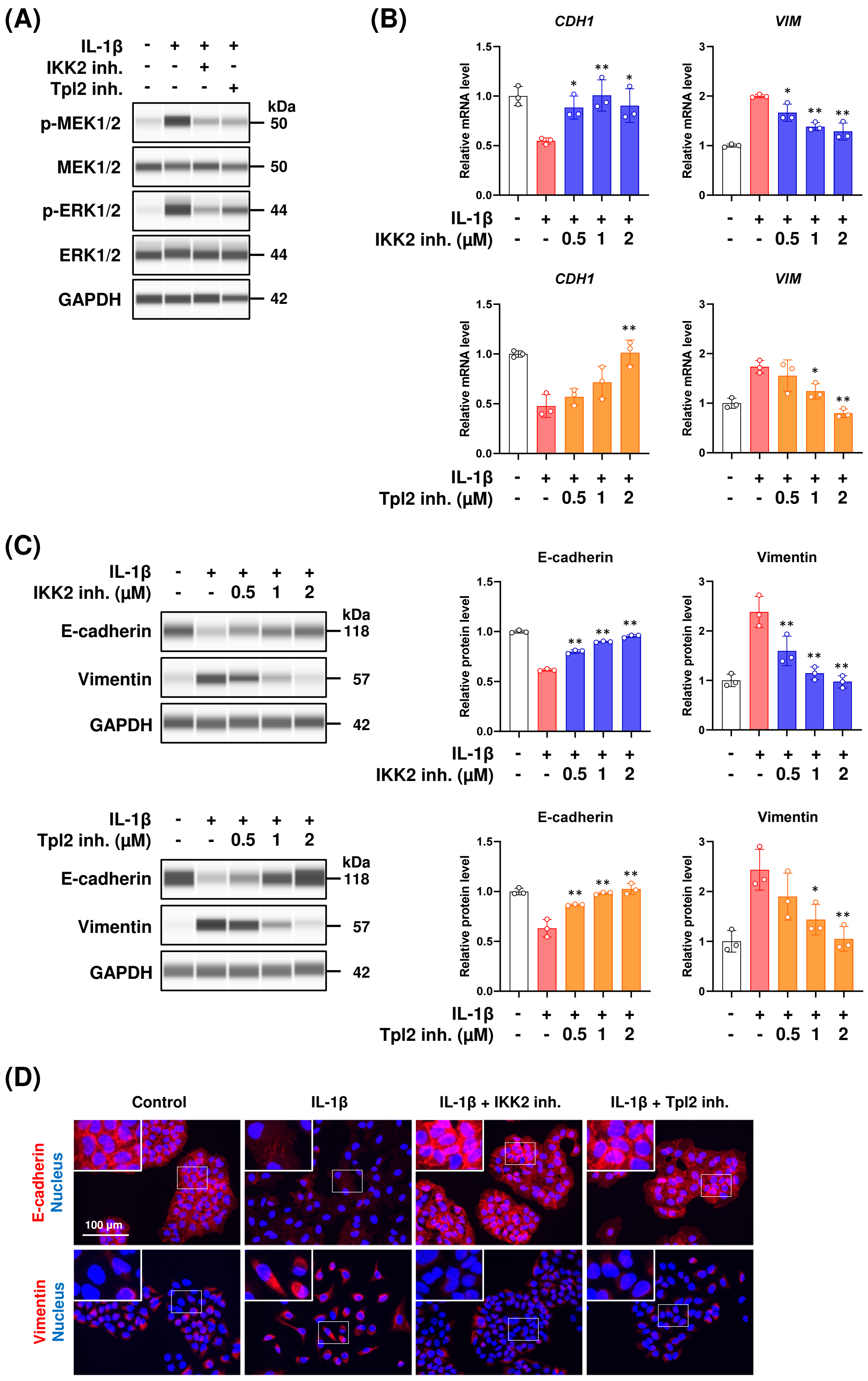

Supplementary Figure S7. Effect of IKK2 inhibitor VI and Tpl2 kinase inhibitor on the induction of EMT in A549 cells stimulated with IL-1β. **A** Western blot analysis of MEK1/2 and ERK1/2 phosphorylation in cells treated with IL-1β in the presence of IKK2 inhibitor VI or Tpl2 kinase inhibitor for 30 min. GAPDH was used as internal control. **B** *CDH1* and *VIM* mRNA expression levels in cells treated with IL-1β and IKK2 inhibitor VI (upper panels) or Tpl2 kinase inhibitor (bottom panels) for 48 h. Each mRNA expression level was normalized to the corresponding *ACTB* value and is presented as relative units to untreated control. Values are means ± SD, n = 3, one-way ANOVA followed by Tukey's multiple comparison test. *P < 0.05, **P < 0.01, compared with IL-1β-treated cells. **C** Western blot analysis of E-cadherin and vimentin in cells treated with IL-1β and IKK2 inhibitor VI (upper panels) or Tpl2 kinase inhibitor (bottom panels) for 48 h. Each protein level was normalized to the corresponding GAPDH value and is presented as relative units to untreated control. Values are means ± SD, n = 4, one-way ANOVA followed by Tukey's multiple comparison test. *P < 0.05, **P < 0.01, compared with IL-1β-treated cells. **D** Immunofluorescence analysis of E-cadherin (upper panels) and vimentin (bottom panels) in cells treated with IL-1β in the presence of IKK2 inhibitor VI or Tpl2 kinase inhibitor for 48 h. Nuclei were stained with NucBlue. Fluorescence images were obtained by using a BZ-X710 fluorescence microscope. Insets show high-magnification images of the boxed areas.

Supplementary Figure S8

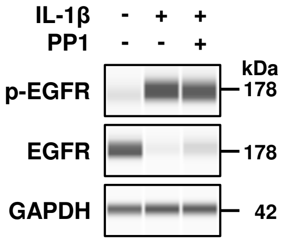

Supplementary Figure S8. Western blot analysis of EGFR phosphorylation in cells stimulated with 100 pg/mL IL-1β in the presence of 2 μM PP1 for 30 min. GAPDH was used as internal control.

Supplementary Table S1

Supplementary Table S1 Primary antibodies used in this study.

	Species	Clone	Dilution	Source
E-cadherin	Rabbit	24E10	1:50 (WB)<br>1:200 (IF)	CST (#3195)
Vimentin	Rabbit	D21H3	1:50 (WB)<br>1:200 (IF)	CST (#5741)
GAPDH	Rabbit	14C10	1:100	CST (#2118)
Phospho-Smad2 (Ser465/467)	Rabbit	138D4	1:50	CST (#3108)
Phospho-Smad3 (Ser423/425)	Rabbit	C25A9	1:50	CST (#9520)
Smad2/3	Rabbit	D7G7	1:50	CST (#8685)
Phospho-NF-κB p65 (Ser536)	Rabbit	93H1	1:50	CST (#3033)
NF-κB p65	Rabbit	D14E12	1:50	CST (#8242)
Phospho-EGFR (Tyr1068)	Rabbit	D7A5	1:50	CST (#3777)
EGFR	Rabbit	D38B1	1:50	CST (#4267)
Phospho-ERK1/2 (Thr202/Tyr204)	Rabbit	D13.14.4E	1:50	CST (#4370)
ERK1/2	Rabbit	137F5	1:50	CST (#4695)
Phospho-AKT (Ser473)	Rabbit	D9E	1:50	CST (#4060)
AKT (pan)	Rabbit	C67E7	1:50	CST (#4691)
Phospho-p38 (Thr180/Tyr182)	Rabbit	D3F9	1:50	CST (#4511)
p38	Rabbit	D13E1	1:50	CST (#8690)
Phospho-JNK (Thr183/Tyr185)	Rabbit	81E11	1:50	CST (#4668)
JNK	Rabbit		1:50	CST (#9252)
Phospho-MEK1/2 (Ser217/221)	Rabbit	41G9	1:50	CST (#9154)
MEK1/2	Rabbit	D1A5	1:50	CST (#8727)

Abbreviations: WB, Western blot; IF, Immunofluorescence; CST, Cell Signaling Technology; EGFR, Epidermal growth factor receptor; NF-κB, Nuclear factor kappa-B; ERK1/2, Extracellular signal-regulated protein kinase 1 and 2; AKT, Protein kinase B; JNK, c-jun N-terminal kinase; MEK, Mitogen-activated and extracellular signal-regulated kinase kinase

Supplementary Table S2

Supplementary Table S2 Chemical inhibitors and neutralizing antibody used in this study.

	Target	Source	Ref.
**Chemical inhibitor**			
BAY11-7082	IκBα Kinase	Abcam (#ab141228)	[1]
AG1478	EGFR tyrosine kinase	Cayman (#10010244)	[2]
PD153035	EGFR tyrosine kinase	Cayman (#18080)	[3]
GM6001	MMP and ADAM family	Cayman (#14533)	[4]
LY294002	PI3K	Cayman (#70920)	[5]
FR180204	ERK	Cayman (#15544)	[6]
SB239063	p38 MAPK	Cayman (#19142)	[7]
SP600125	JNK	Cayman (#10010466)	[8]
PD98059	MEK	Cayman (#10006726)	[9]
IKK2 inhibitor VI	IKKβ	Cayman (#17276)	[10]
Tpl2 kinase inhibitor	Tpl2 kinase	Cayman (#19710)	[11]
PP1	Src family	Cayman (#14244)	[12]
**Neutralizing antibody**			
Anti-EGFR antibody (clone LA1)	EGFR	Sigma-Aldrich (#05-101)	[13]

Abbreviations: IκBα, Inhibitor of kappa-B α; EGFR, Epidermal growth factor receptor; MMP, Matrix metalloproteinase; ADAM, A disintegrin and metalloproteinase; PI3K, Phosphoinositide 3-kinase; ERK, Extracellular signal-regulated protein kinase; p38 MAPK, p38 mitogen-activated protein kinase; JNK, c-jun N-terminal kinase; MEK, Mitogen-activated and extracellular signal-regulated kinase kinase; IKKβ, IκB kinase β; Tpl2, Tumor progression locus 2

Supplementary References

1. Pierce JW, Schoenleber R, Jesmok G, Best J, Moore SA, Collins T, et al. Novel inhibitors of cytokine-induced IκBα phosphorylation and endothelial cell adhesion molecule expression show anti-inflammatory effects *in vivo*. J Biol Chem. 1997;272:21096-103.
2. Osherov N, Levitzki A. Epidermal- growth-factor-dependent activation of the src-family kinases. Eur J Biochem. 1994;225:1047-53.
3. Fry DW, Kraker AJ, McMichael A, Ambroso LA, Nelson JM, Leopold WR, et al. A specific inhibitor of the epidermal growth factor receptor tyrosine kinase. Science. 1994;265:1093-5.
4. Grobelny D, Poncz L, Galardy RE. Inhibition of human skin fibroblast collagenase, thermolysin, and Pseudomonas aeruginosa elastase by peptide hydroxamic acid. Biochemistry. 1992;31:7152-4.
5. Vlahos CJ, Matter WF, Hui KY, Brown RF. A specific inhibitor of phosphatidylinositol 3-kinase, 2-(4-morpholinyl)-8-phenyl-4H-1-benzopyran-4-one (LY294002). J Biol Chem. 1994;269:5241-8.
6. Ohori M, Kinoshita T, Okubo M, Sato K, Yamazaki A, Arakawa H, et al. Identification of a selective ERK inhibitor and structural determination of the inhibitor-ERK2 complex. Biochem Biophys Res Commun. 2005;336:357-63.
7. Underwood DC, Osborn RR, Bochnowicz S, Webb EF, Rieman DJ, Lee JC, et al. SB239063, a p38 MAPK inhibitor, reduces neutrophilia, inflammatory cytokines, MMP-9, and fibrosis in lung. Am J Physiol Lung Cell Mol Physiol. 2000;279:L895-902.
8. Bennett BL, Sasaki DT, Murray BW, O'Leary EC, Sakata ST, Xu W, et al. SP600125, an anthrapyrazolone inhibitor of Jun N-terminal kinase. Proc Natl Acad Sci U S A. 2001;98:13981-6.
9. Alessi DR, Cuenda A, Cohen P, Dudley DT, Saltiel AR. PD 98059 is a specific inhibitor of the activation of mitogen-activated protein kinase kinase *in vitro* and *in vivo*. J Biol Chem. 1995;270:27489-94.
10. Baxter A, Brough S, Cooper A, Floettmann E, Foster S, Harding C, et al. Hit-to-lead studies: the discovery of potent, orally active, thiophenecarboxamide IKK-2 inhibitors. Bioorg Med Chem Lett. 2004;14:2817-22.
11. Gavrin LK, Green N, Hu Y, Janz K, Kaila N, Li HQ, et al. Inhibition of Tpl2 kinase and TNF-alpha production with 1,7-naphthyridine-3-carbonitriles: synthesis and structure-activity relationships. Bioorg Med Chem Lett. 2005;15:5288-92.
12. Hanke JH, Gardner JP, Dow RL, Changelian PS, Brissette WH, Weringer EJ, et al. Discovery of novel, potent, and Src family-selective tyrosine kinase inhibitor. Study of Lck- and FynT-dependent T cell activation. J Biol Chem. 1996;271:695-701.
13. Asano K, Nakamura H, Lilly CM, Klagsbrun M, Drazen JM. Interferon gamma induces prostaglandin G/H synthase-2 through an autocrine loop via the epidermal growth factor receptor in human bronchial epithelial cells. J Clin Invest. 1997;99:1057-63.
